# Bumblebees mediate landscape effects on a forest herb's population genetic structure in European agricultural landscapes

**DOI:** 10.1002/ece3.70078

**Published:** 2024-07-25

**Authors:** Jannis Till Feigs, Siyu Huang, Stephanie I. J. Holzhauer, Jörg Brunet, Martin Diekmann, Per‐Ola Hedwall, Katja Kramp, Tobias Naaf

**Affiliations:** ^1^ Leibniz Centre for Agricultural Landscape Research (ZALF) e.V Müncheberg Germany; ^2^ Thünen Institute of Biodiversity, Johann Heinrich von Thünen Institute, Forestry and Fisheries, Federal Research Institute for Rural Areas Braunschweig Germany; ^3^ Southern Swedish Forest Research Centre Swedish University of Agricultural Sciences Alnarp Sweden; ^4^ Vegetation Ecology and Conservation Biology, Institute of Ecology, FB 2 University of Bremen Bremen Germany

**Keywords:** bumblebees, forest herbs, genetic linker, genetic structure, landscape composition, landscape genetics, SSR

## Abstract

Spatially isolated plant populations in agricultural landscapes exhibit genetic responses not only to habitat fragmentation per se but also to the composition of the landscape matrix between habitat patches. These responses can only be understood by examining how the landscape matrix influences among‐habitat movements of pollinators and seed vectors, which act as genetic linkers among populations. We studied the forest herb *Polygonatum multiflorum* and its associated pollinator and genetic linker, the bumblebee *Bombus pascuorum*, in three European agricultural landscapes. We aimed to identify which landscape features affect the movement activity of *B. pascuorum* between forest patches and to assess the relative importance of these features in explaining the forest herb's population genetic structure. We applied microsatellite markers to estimate the movement activity of the bumblebee as well as the population genetic structure of the forest herb. We modelled the movement activity as a function of various landscape metrics. Those metrics found to explain the movement activity best were then used to explain the population genetic structure of the forest herb. The bumblebee movement activity was affected by the cover of maize fields and semi‐natural grasslands on a larger spatial scale and by landscape heterogeneity on a smaller spatial scale. For some measures of the forest herb's population genetic structure, that is, allelic richness, observed heterozygosity and the *F*‐value, the combinations of landscape metrics, which explained the linker movement activity best, yielded lower AICc values than 95% of the models including all possible combinations of landscape metrics.

Synthesis: The genetic linker, *B. pascuorum*, mediates landscape effects on the population genetic structure of the forest herb *P. multiflorum*. Our study indicates, that the movement of the genetic linker among forest patches, and thus the pollen driven gene flow of the herb, depends on the relative value of floral resources in the specific landscape setting. Noteworthy, the population genetic structure of the long‐lived, clonal forest herb species correlated with recent land‐use types such as maize, which have been existing for not more than a few decades within these landscapes. This underscores the short time in which land‐use changes can influence the evolutionary potential of long‐lived wild plants.

## Graphical abstract

Bumblebees can connect isolated populations of forest herbs within agricultural landscapes by moving between forest patches. These movements are influenced by specific aspects of the landscape. These same landscape aspects also partly explain the population genetic structure of a forest herb species associated with bumblebees as pollinators.

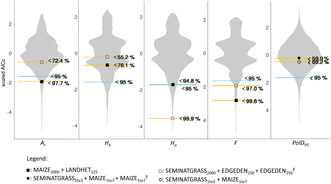

## 1 INTRODUCTION

Numerous specialist species evolved under long‐term stable conditions within extensive areas of contiguous habitat in late successional states, such as forests (Pickett, 1976). However, human activities transformed such landscapes significantly in recent centuries. Large parts once dominated by natural habitats were converted into agricultural landscapes (Ellis, 2011). These landscapes are composed of mosaics of various land‐use types, with semi‐natural habitat patches being small and isolated from each other (Hendrickx et al., 2007; Kennedy et al., 2019). Additionally, agriculture itself is continuously evolving, resulting in changes in farming practices and crop types grown (Thrall et al., 2010). Besides direct effects of habitat loss and isolation, wild plant populations are indirectly affected by how the landscape composition impacts their genetic linkers, that is, the animals that connect distinct plant populations by transporting pollen or seeds (Feigs et al., 2022; Jeltsch et al., 2013). For these mobile organisms, there are diverse mechanisms by which the landscape composition influences their abundance and movement behaviour. This can occur by offering nesting or foraging habitats (Miller & Cale, 2000; Westrich, 1996), and by guiding or hindering passage between habitats (Klaus et al., 2015; Krewenka et al., 2011). The effects of landscape composition on the movement of these linkers among plant populations play a crucial role in determining their genetic connectivity (Aguilar et al., 2006).

The species‐rich herb layer of temperate forest patches contributes a relevant proportion to the overall biodiversity within agricultural landscapes (Billeter et al., 2008; Valdés et al., 2015). Many forest herb species exhibit traits that can be interpreted as adaptions to long‐term stable conditions, that is, they employ clonal reproduction strategies and produce few and heavy seeds, making long‐distance seed dispersal a rare event (Honnay et al., 2005; Whigham, 2004). If populations of such species are small and isolated, genetic linkers that realize gene flow across the agricultural matrix should be essential for their long‐term survival (Honnay et al., 2005; Young et al., 1996). Theoretically, forest herb populations could be buffered from the effects of landscape composition changes in the short term by primarily reproducing vegetatively (Honnay et al., 2005), but numerous studies have demonstrated that habitat loss and fragmentation affect the population genetic structure of forest herbs within agricultural landscapes (Gentili et al., 2018; Jacquemyn et al., 2006; Kolb & Durka, 2013; Naaf et al., 2021; Vandepitte et al., 2007; Vellend, 2004). Furthermore, studies have suggested that the landscape composition in between the forest patches may also shape the herbs' population genetic structure (Decocq et al., 2021; Guiller et al., 2023; Schmidt et al., 2009). In fact, the landscape composition might even exert a greater influence on the population genetic structure of forest herbs than habitat loss and fragmentation per se (Naaf et al., 2022). Review articles for various types of mosaic‐like landscapes highlight that landscape composition is a major determinant of functional connectivity of plant populations (Driscoll et al., 2013; Murphy & Lovett‐Doust, 2004). The presumed mechanism here is the influence of the landscape composition on the behaviour of the genetic linkers. Therefore, landscape effects on plants were often interpreted as responses of seed or pollen vectors to the landscape composition (Aavik et al., 2017; Favre‐Bac et al., 2016; Kamm et al., 2010; Kramer et al., 2011; Schmidt et al., 2009). Occasionally, genetic linkers are also invoked to explain the absence of genetic isolation effects among spatially fragmented plant populations (Honnay et al., 2006; Sork & Smouse, 2006).

For forest herb species with low seed dispersal capabilities, the effects of specific landscape elements and the overall composition of the landscape on the movement activity of associated pollinators might be particularly relevant. Such effects of landscape composition on abundances and behaviour have been shown for various pollinators including bees (Krewenka et al., 2011), flies (Haenke et al., 2014), butterflies (Flick et al., 2012) and birds (Tscharntke et al., 2008). One type of pollinator that has been shown to act as a genetic linker among forest herb populations is the foraging workers of bumblebees (Feigs et al., 2022). Bumblebees pollinate a range of forest herbs, especially species with long corollas, such as *Polygonatum* spp. (Hasegawa & Kudo, 2005; Naaf et al., 2021), *Phyteuma* spp. (Kolb, 2008), Primula spp. (Van Rossum et al., 2011) or *Stachys sylvatica* (Fussell & Corbet, 1991). They can move over longer distances and regularly traverse the agricultural matrix (Redhead et al., 2016). The abundance of bumblebee workers, as well as their foraging or nesting behaviour, has been shown to respond to landscape elements, such as different crop types or linear elements. For instance, rapeseed cover at a landscape scale was positively related to bumblebee density (Westphal et al., 2003) as well as colony growth (Westphal et al., 2006). Whether these effects of rapeseed translate into increasing or decreasing pollination services in nearby semi‐natural habitats depends on the spatial scale considered (Kovács‐Hostyánszki et al., 2013). Other studies have shown that bumblebees tend to avoid crossing linear landscape elements, such as hedgerows (Cranmer et al., 2012; Garratt et al., 2017; Klaus et al., 2015) and roads (Bhattacharya et al., 2003), and instead prefer flying along them. Beyond specific landscape elements, landscapes featuring a higher diversity of land‐use types are considered to provide more abundant and diverse floral resources for pollinators (Persson & Smith, 2013). This enhanced food supply resulted in larger body sizes of bumblebee workers (Persson & Smith, 2011), which could indicate larger foraging ranges (Grass et al., 2018), potentially increasing their activity as a genetic linker. However, bumblebees exhibited shorter foraging distances in more complex landscapes compared to more homogeneous landscapes (Jha & Kremen, 2013), such as those dominated by a single crop like maize (Hass et al., 2019).

However, studies focusing on the effects of landscape composition on pollinators do not typically address their role as genetic linkers for particular plants within specific habitats. From the plant's perspective, it is not the pollinator's abundance and general movement activity across the landscape that is relevant for gene flow, but rather it is directed movement between the plant's populations in distinct habitat patches (Hadley & Betts, 2012). An assessment of the impact of landscape composition on the capacity of pollinators to function as genetic linkers is therefore needed. Such an integrated approach may show that landscape effects on the genetic linker's movement activity among habitat patches translate into landscape effects on the population genetic structure of the associated plant species. Only a few studies have shown that landscape effects on pollinators could be translated into landscape effects on plants (Cranmer et al., 2012; Herbertsson et al., 2021; Meyer et al., 2005), but these scarce cases provide good examples of how to address the plant–pollinator–landscape complex in a more holistic study design. They demonstrated how higher seed sets occur as a consequence of landscape effects on pollen vector activity. Unaddressed remains, however, how pollinator movements translate into realized gene flow among spatially isolated plant populations. Some studies tackled this question using fluorescent dye (Kormann et al., 2016; Van Geert et al., 2014; Van Rossum et al., 2011). Their analyses provided a comprehensive understanding of the impacts of the distances covered by all pollen vectors and the quantity of pollen they convey. However, different pollinator species respond distinctively to the landscape, and the realized pollen flow reflects the combined outcome of all these interactions. The same is true for studies that analysed the effects of communities of pollinators with varying mobility on the population genetic structure of plants (Castilla et al., 2017; Torres‐Vanegas et al., 2019). Consequently, the precise contribution of a particular pollinator species to gene flow remains undisclosed by those approaches.

To address effectively how a specific genetic linker mediates landscape effects on the genetic structure of plant populations, a study should combine landscape data, movement data of the genetic linker and population genetic structure data of the plant species. Previous studies demonstrated that the recent agricultural landscape composition significantly affects the genetic diversity and differentiation of populations of the forest herb *Polygonatum multiflorum* despite its longevity (Naaf et al., 2022) and that this genetic diversity and differentiation is also significantly affected by the movement activity of one of *P. multiflorum's* main pollinators, that is, *Bombus pascuorum* (Feigs et al., 2022). With the present study, we now aim (a) to investigate how the landscape composition affects the movement activity of *B. pascuorum* as a genetic linker of *P. multiflorum*, and (b) to assess how much of the forest herb's population genetic structure can be explained by those landscape features that influence the movement activity of *B. pascuorum*. We examined these two objectives by testing the following two hypotheses:

The landscape composition around the forest patches and between pairs of forest patches significantly affects the movement activity of the genetic linker *B. pascuorum*.

Those landscape metrics that are most relevant to explain the bumblebee's movement activity will also contribute significantly to explaining the population genetic structure of the forest herb. In particular, these landscape metrics will explain the herb's population genetic structure better than random combinations of landscape metrics.

## 2 MATERIALS AND METHODS

### 2.1 Study species

*Polygonatum multiflorum* (L.) ALL. (Figure S1A–C) is a slow‐colonizing forest specialist (Brunet, 2007; Schmidt et al., 2014; Verheyen et al., 2003) which exhibits not only strong clonal growth but also regular seedling recruitment (Kosiński, 2012). The species blooms in spring, is strictly outcrossing and depends on insect pollination (Klotz et al., 2002). Its flowers grow on axillary peduncles, with 2–6 flowers in the leaf axils, and blossom sequentially from the top to the bottom of the shoot (Kosiński, 2012). Its corolla is specialized in long‐tongued bumblebees as pollinators (Feigs et al., 2022; Kosiński, 2012; Naaf et al., 2021).

Our own field observations showed that, among the identified species, the two bumblebee species *B. pascuorum* and *B. pratorum* contributed approximately 93% (54% for *B. pascuorum* and 39% for *B. pratorum*) of pollination events during 53.5 h of flower observation for *P. multiflorum* in isolated forest patches within European agricultural landscapes. *Bombus pascuorum* (SCOPOLI, 1763) (Figure S1C) is one of the most common long‐tongued bumblebee species in European landscapes including fields and forests (Gómez‐Martínez et al., 2020). Similar to other bumblebee species, *B. pascuorum* is a central‐place forager with a queen establishing a nest in spring at a suitable position, which is often found along the boundaries between the field and the forest (Kells & Goulson, 2003). From this central position, workers fly to foraging habitats with floral resources, which in the case of *B. pascuorum* include forests. It was shown that the workers' movement activity of *B. pascuorum* is correlated with the population genetic structure of *P. multiflorum* (Feigs et al., 2022). Knowledge regarding the seed vectors of *P. multiflorum* is limited, but it is considered to have a low seed dispersal potential and is classified as autochorous (Müller‐Schneider, 1986). Long‐distance dispersal of its toxic fleshy berries by birds or mid‐sized carnivores is considered rare (Ehrlén & Eriksson, 2000; Müller‐Schneider, 1986; Schaumann & Heinken, 2002), while short‐distance dispersal by rodents might happen more frequently (Ehrlén & Eriksson, 1993).

### 2.2 Landscape analysis

This study was conducted in three 5 x 5 km landscape windows within typical Central European agricultural landscapes, located in western Germany, eastern Germany and southern Sweden. The three landscape windows differed slightly in their landscape composition (Supplement S2). In each landscape window, we selected six forest patches that were occupied by *P. multiflorum* and that were forested at least since the 19th century. We considered the individuals of *P. multiflorum* within one forest patch as a distinct population. The population boundary did not necessarily align with the forest patch boundary. In this case, we checked if no further individuals were present within a 100‐m buffer. We analysed the landscape in between these forest patches at two levels (Naaf et al., 2022): the node level with buffer zones around each forest herb population (Schmidt et al., 2009) and the link level with rectangular landscape strips connecting the centres of each plant population (Braunisch et al., 2010). To do so, we created digital land‐use maps with ESRI ArcGIS Map version 10.8.2 (Figure S2) for the three landscape windows based on recent orthophotos according to Naaf et al. (2022). For arable fields, we also differentiated the dominance of three different crop types, that is, oil seed rape, maize and other cereals, over the preceding decade (Figures S2.3–S2.5). The underlying data were collected within the European Integrated Administration and Control System (IACS) (European Commission 2020). Crop‐type dominance was measured as raster data with a cell size of 10 m. One dominance value was calculated for each cell for a period from 2008 (eastern Germany, southern Sweden) and 2009 (western Germany) to 2017 for the forest herbs and from 2008/2009 to 2019 for the genetic linker. A dominance value of 1 indicates the presence of the crop type in each year and a value of 0 indicates absence across all years. The dominance values for both periods were highly correlated (Figures S2.6–S2.7). We calculated the per cent cover of 11 area‐based land‐use types, the relative length of 4 linear landscape elements (= total length divided by the area of the buffer zone or the strip area, respectively) and 2 index measures, that is, the Shannon diversity of land‐use types, from here on called landscape heterogeneity, and the density of all land‐use patch edges (Table 1). We used five different buffer distances (125 , 250, 500, 1000 and 2000 m) at the node level and five different width‐to‐length ratios for the landscape strips (1:7, 1:5, 1:3, 1:2 and 2:3) at the link level.

**TABLE 1:** We used 11 area‐based landscape metrics, 4 linear landscape elements and 2 index measures.

Area‐based metrics	Per cent cover of…
D_FOREST	Deciduous forest
C_FOREST	Coniferous forest
GRASS	Grassland in general
SEMNATGRASS	Semi‐natural grassland
SEMNATVEG	Other semi‐natural vegetation
ORCHARD	Traditional orchards
SETTLE	Settlement area
ARABLE	Arable land in general (includes also rapeseed, maize and cereal)
RAPESEED	Oilseed rape
MAIZE	Maize
CEREAL	Cereals

Linear landscape elements	Relative length of…
L_FRINGE	Herbaceous fringes (<3 m width)
L_WOOD	Woody linear elements
L_ROAD	Roads
L_WATER	Water courses
**Index metrics**	
LANDHET	Landscape heterogeneity (Shannon diversity of land‐use types)
EDGEDEN	Land‐use parcel edge density [m ha ^−1^]

*Note*: All land‐use variables were measured at the node and at the link level in five buffer distances /width‐to‐length ratios (node level: 125, 250, 500, 1000 and, 2000 m; link level: 1:7, 1:5, 1:3, 1:2 and, 2:3).

### 2.3 Sampling and genotyping

Within the selected 18 forest patches, we sampled leaf material of 20 individuals per *P. multiflorum* population in spring of 2018 (Table S4.1). Less than 20 individuals were used when population sizes were very small or genotyping failed (Table S4.1). Additionally, we collected 14–36 (mean = 24) individuals of *B. pascuorum* (Table S4.1) in each of these forest patches during spring in 2018 and 2019. We collected the bumblebees with Malaise traps that were placed in the middle of flowering patches of *P. multiflorum* (Figure S1B) and with hand net catches. We used microsatellite markers to estimate (a) the population genetic structure of *P. multiflorum* and (b) the movement activity of *B. pascuorum*. The marker set of *P. multiflorum* consisted of 6 loci, resulting in 134 alleles, while the marker set of *B. pascuorum* comprised 8 loci, yielding 148 alleles. Both marker sets could successfully distinguish between the sampled individuals. For detailed information regarding DNA extraction, primers, PCR conditions, genotyping error rates and checks for the occurrence of clones, see Naaf et al. (2021) for *P. multiflorum* and Feigs et al. (2022) for *B. pascuorum*.

### 2.4 Indicators of population genetic structure and movement activity

At the node level, we calculated four genetic measures for *P. multiflorum*, including allelic richness (*A*_*r*_), expected heterozygosity (*H*_*e*_), observed heterozygosity (*H*_*o*_) and the *F*‐value (*F* = *H*_*e*_/*H*_*o*_). For clonal plant species in small and fragmented populations, the *F*‐value can deviate negatively from Hardy–Weinberg equilibrium (Stoeckel et al., 2006). A Previous study found such heterozygote excess for *P. multiflorum* with significant negative *F*‐values (Feigs et al., 2022). Here, observed heterozygosity per population was larger than expected heterozygosity. In this case, the *F*‐value cannot be interpreted as an *inbreeding coefficient* (Stoeckel et al., 2006). Instead, we anticipate that higher gene flow among populations leads to an *F‐value* closer to zero and a decrease in observed heterozygosity.

At the link level, we used 1 minus the pairwise proportion of shared alleles (*D*_PS_). In a previous study (Feigs et al., 2022), they found *D*_PS_ of *P. multiflorum*, from here on called *PolD*_PS_, to be positively affected by the bumblebee movement activity.

We used genetic measures also to estimate the movement activity of *B. pascuorum*. Genetic analysis is an effective method for estimating the movement activity of flying insects, especially when high numbers of individuals and larger ranges are included in the analysis (Goulson, 2010; Osborne et al., 2002). However, not all forms of a pollinator's movement are equally relevant for serving as a genetic linker (Jeltsch et al., 2013). One relevant type is the foraging movement of bumblebee workers. This is because the workers of the same nest communicate about forage resources (Dornhaus & Chittka, 1999), are relatively flower constant (Chittka et al., 1999; Goulson, 2010) and establish fixed trap lines (Ohashi & Thomson, 2009). To estimate the foraging movement activity of bumblebee nests, an effective framework utilizes sibship assignment (Carvell et al., 2012; Jha & Kremen, 2013; Knight et al., 2005; Redhead et al., 2016). Here, workers from different locations are used to estimate nest‐specific foraging distances, similar to mark–recapture studies, but with putative siblings as reobserved units (Mola & Williams, 2019). In this study, we apply the same rationale but analyse which nests contribute workers to multiple forest patches. For each forest patch, we computed (a) *NESTS*_*shared*_: the number of nests assigned to focal forest patch *i* that are shared with at least one other forest patch, divided by the total number of nests assigned to forest patch *I*; and (b) *FOREST‐PATCHES*_*shared*_: the number of forest patches with which focal forest patch *i* shares at least one assigned nest. To calculate the two indicators at the node level, we identified which workers of *B. pascuorum* shared the same nest by using the full‐likelihood algorithm of the COLONY 2.0 software (Jones & Wang, 2010). We ran the software with the settings ‘monogamous mating’ for both males and females and a ‘medium long run’, following the settings published for the same species in Dreier et al. (2014). Each combination of landscape window and year was analysed separately. The runs were repeated with different random numbers of seeds. If the probability of individuals being full siblings was larger than 80% in both runs, we treated them as individuals from a shared nest (Feigs et al., 2022).

The two indicators for the movement activity among‐forest patches at the node level have been identified as relevant for the gene flow of *P. multiflorum* among‐forest patches in a previous study (Feigs et al., 2022). Another movement indicator, which has been found to be relevant for the gene flow of *P. multiflorum* at the link level, was *D*_PS_ (1 minus the pairwise proportion of shared alleles) for *B. pascuorum*, from here on called *BomD*_PS_ to avoid confusion with *PolD*_PS_. Unlike the indicators based on nest estimation, this measure partially reflects other movement types besides the workers' foraging activities as the dispersal of the species in the landscape window. All three of them are indirect indicators derived from the relationship of specimens and their sampling locations.

### 2.5 Data analysis

In 17 of the 18 studied forest patches, the number of captured individuals of *B. pascuorum* was >10, which we considered sufficient for our analyses (Table S4.1). We employed linear mixed models (LMM) using the *lme* function from the R package *nlme* version 3.1‐155 (Pinheiro et al., 2019). In the link‐level analysis, we incorporated the dependency structure of plant population pairs that shared a common population. This was achieved by defining the correlation structure using the *corMLPE* function (Pope, 2020). We also added the geographic distance between the centres of the plant populations as a potential explanatory variable besides the landscape metrics, since a previous study showed that it might be relevant for explaining genetic differentiation of *P. multiflorum* (Naaf et al., 2021). To improve the symmetry of the variable distributions, all variables were Box‐Cox transformed. Subsequently, they were centred and scaled to mean = 0 and standard deviation = 1 to obtain standardized regression coefficients. The landscape windows were included in all models as a random intercept term.

**FIGURE 1:**
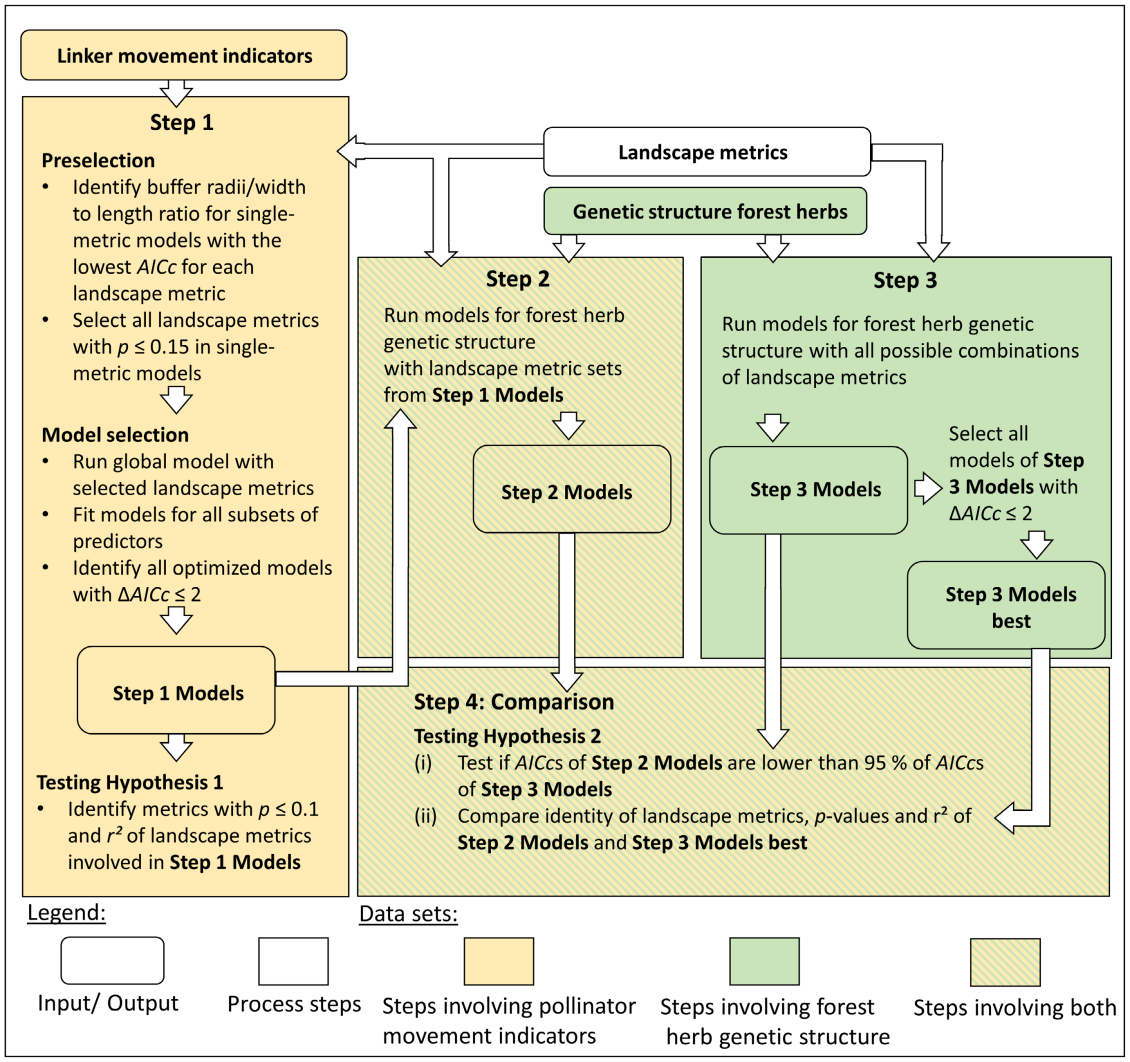
Overview of the four steps of data analysis. Input/output and process steps are depicted using different shapes. The colour of the objects indicates which data sets are involved in each specific step.

We conducted our analysis in four steps, as illustrated in Figure 1.

*Step 1* served two purposes, that is, to test our hypothesis 1 on the linker's movement activity and to identify those sets of landscape metrics that will be used for modelling the herb's population genetic structure in Step 2. This step involved modelling the linker's movement activity indicators (*NESTS*_*shared*_, *FOREST‐PATCHES*_*shared*_ and *BomD*_PS_) as a function of landscape metrics. These models are referred to as *Step 1 Models* (Figure 1). First, we determined at which buffer size (node level) or width‐to‐length ratio (link level) each landscape metric showed the strongest effect. We selected the models with buffer distances or width‐to‐length ratios that yielded the lowest AICc (Akaike information criterion corrected for small sample size; Anderson & Burnham, 2002) for each landscape metric individually. To account for curvilinear or unimodal relationships, quadratic terms were included in the models if they lowered the AICc. In the subsequent steps, we focused only on landscape metrics that showed an effect in the single‐metric models at a significance level of alpha = 0.15 based on likelihood‐ratio tests. This relatively tolerant threshold was used as a preselection criterion for the following multivariable models to avoid excluding potentially important candidates. Second, we applied model selection by fitting all subsets of the remaining landscape metrics, with a maximum of two terms at the node level and four terms at the link level. Landscape metrics with a Pearson correlation of |*r*| ≥ .7 were not used simultaneously in the same model. We kept all models with ∆AICc < 2 and used the results to answer Hypothesis 1, interpreting all effects with *p* < .1 in the selected models. Since the landscape metrics do not only reflect the landscape composition in the sampling years but also the recent past, we believe that the detected effects on movement activity among‐forest patches should be consistent across different years. The corresponding sets of landscape metrics were used for further analyses in Step 2.

In *Step 2*, we modelled the forest herb's population genetic structure as a function of those sets of landscape metrics that were identified as most relevant for the pollinator movement activity in Step 1. These models are referred to as *Step 2 Models* (Figure 1). At the node level, the genetic response variables were *A*_*r*_, *H*_*e*_, *H*_*o*_ and *F*; at the link level: *PolD*_PS_. Statistical significance of model terms was assessed with *t*‐tests.

The purpose of *Step 3* was to model the population genetic structure of the forest herb (node level: *A*_*r*_, *H*_*e*_, *H*_*o*_ and *F*; link level: *PolD*_PS_) as a function of all possible combinations of landscape metrics including all radii and length‐to‐width ratios. These models are referred to as *Step 3 Models* (Figure 1). The Step 3 Models included all possible combinations of linear and quadratic terms. A quadratic term was only allowed if its linear term was also included in the same model. At the node level, two terms were allowed, and at the link level, four terms were allowed, according to the respective sample sizes. We excluded models that involved combinations of landscape metrics with a collinearity of |*r*| ≥ .7 and models that the *lme* function was unable to fit due to convergence failures. All models of Step 3 Models with ∆AICc < 2 were selected as *Step 3 Models best*.

Finally, in *Step 4*, we compared the outcomes of *Step 2 Models* with those of *Step 3 Models*. The rationale behind this is to validate to which extent effects of landscape metrics on the genetic structure of the forest herb are mediated by *B. pascuorum's* movement activity. The population genetic structure of the forest herb reflects the sum of all historical and recent effects of pollinators and seed vectors, as well as other demographic processes. This is particularly true for *P. multiflorum*, given its' longevity and overlapping generations. The herb's genetic structure will react more slowly to the landscape composition than the linker's genetic movement indicators. With these conditions in mind, it is essential for our study to separate the signal that landscape metrics left in the population genetic structure of the forest herb via the pollinator's movement activity from other causes, and to ensure that any significant effects are not a result of chance. We achieve this through two complementary approaches:

1. For each population genetic measure, we tested whether the AICc values of Step 2 Models, including the landscape metrics selected for the genetic linker, were lower than the AICc values of 95% of Step 3 Models, which encompassed the complete set of landscape metrics, thereby accounting for all potential causes of the population genetic structure. If this was the case, we interpreted it as a signal that the genetic linker's movement activity contributes to the detectable landscape effects on the forest herb's population genetic structure.
2. We also compared the goodness of fit of Step 2 Models and Step 3 Models best, as well as the identity of included landscape metrics and their effect sizes and directions, in a descriptive way.

## 3 RESULTS

### 3.1 Landscape effects on pollinator movement activity (Step 1 Models)

**FIGURE 2:**
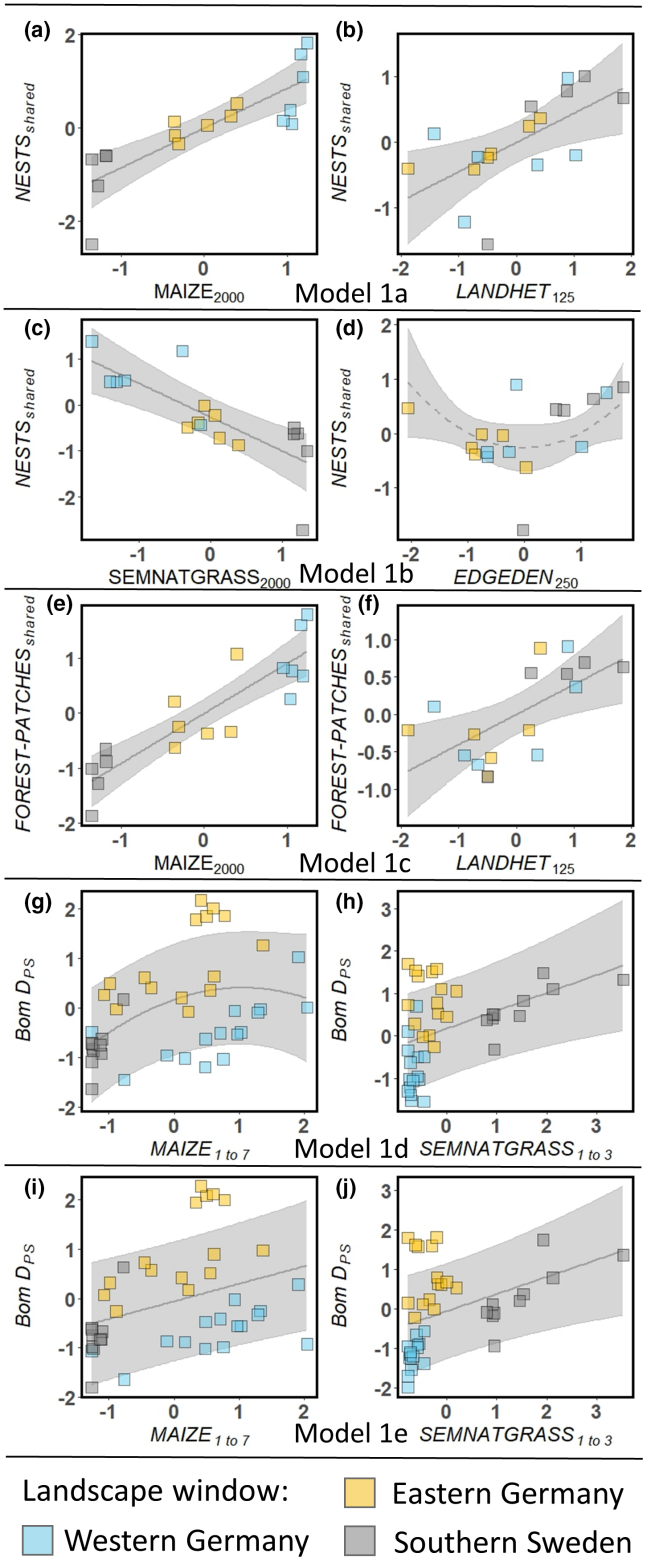
Visualization of landscape effects (cf. Table 2) on indicators of *B. pascuorum* movements among‐forest patches (Step 1 Models). The figures present the partial effects of the different Step 1 Models 1a–1e with significance levels of *p* < .05 (depicted as solid lines) and *p* < .1 (depicted as dashed lines). The 95% confidence bands are represented in grey, and the filled squares represent the partial residuals.

In the multimetric models and at the node level, there were two best models with ∆AICc < 2 for *NESTS*_*shared*_ (Table 2: Models 1a and 1b) and a single best one for *FOREST‐PATCHES*_*shared*_ (Model 1c). In Models 1a and 1c maize cover in a buffer distance of 2000 m (Figure 2a,e) and landscape heterogeneity within 125 m buffer distance (Figure 2b,f) showed positive effects on *NESTS*_*shared*_ and on *FOREST‐PATCHES*_*shared*_. In Model 1b, *NESTS*_*shared*_ decreased with per cent cover of semi‐natural grassland within a 2000 m buffer distance (Figure 2c). Edge density within a 250 m buffer distance had an unimodal effect that was marginally significant (*p* = .0519) (Figure 2d). At the link level, there were two best models for *BomD*_PS_. The effects of both landscape metrics on *BomD*_PS_ were positive (Figure 2g–j).

**TABLE 2:** Summary of five Step 1 Models (landscape effects on pollinator movement indicators) at the node level (*NESTS*_*shared*_, *FOREST‐PATCHES*_*shared*_) and link level (*BomD*_PS_).

Models	Movement indicator	Included landscape metrics with regression coefficient and *p*‐value			*r* ^*2*^
Model 1a	*NESTS*_*shared*_	**MAIZE2000**	***LANDHET125***		.66/.66
		*b* = 0.85, *p* = .0001	*b* = 0.45, *p* = .0143		
Model 1b	*NESTS*_*shared*_	**SEMNATGRASS2000**	**EDGEDEN250**	**EDGEDEN250** ^**2**^	.66/.66
		*b* = −0.78, *p* = .0003	*b* = 0.24, *p* = .1473	*b* = 0.28, *p* = .0519	
Model 1c	*FOREST‐PATCHES*_*shared*_	***MAIZE2000***	***LANDHET125***		.75/.75
		*b* = 0.92, *p* < .0001	*b* = 0.4, *p* = .012		
Model 1d	*BomD*_PS_	**SEMNATGRASS1to3**	**MAIZE1to7**	**MAIZE1to7** ^**2**^	.09/.72
		*b* = 0.42, *p* = .0104	*b* = 0.45, *p* = .0007	*b* = −0.21, *p* = .0493	
Model 1e	*BomD*_PS_	**SEMNATGRASS1to3**	**MAIZE1to7**		.08/.74
		*b* = 0.43, *p* = .0110	*b* = 0.36, *p* = .0043		

*Note*: Shown are the included landscape metrics for each model, the marginal/conditional *r* ^*2*^ values and standardized regression coefficients *b* and *p*‐values.

### 3.2 Translating landscape effects to the forest herb (Step 2 Models)

**FIGURE 3:**
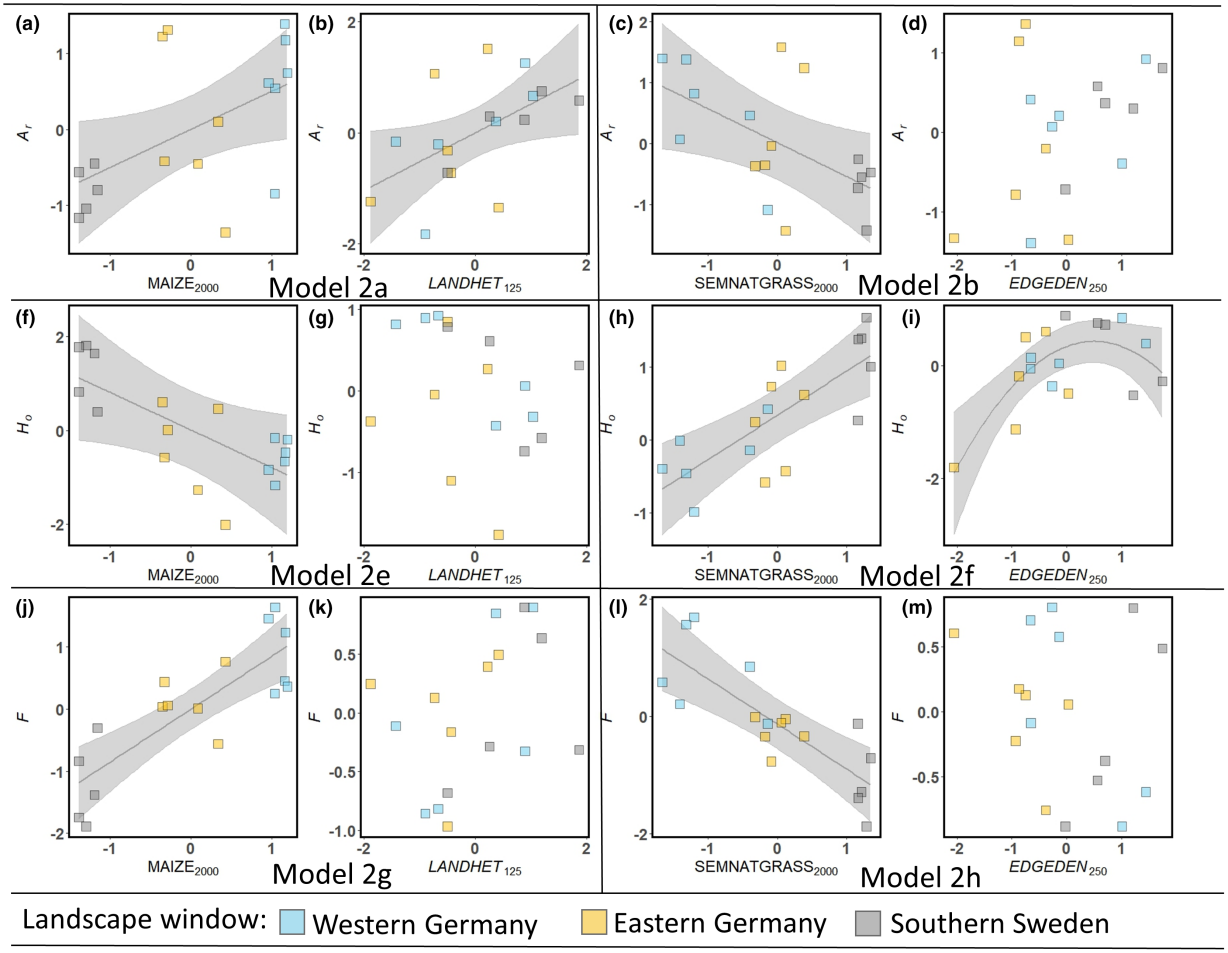
Visualization of landscape effects (cf. Table 3) on the population genetic structure of *Polygonatum multiflorum* (Step 2 Models) based on sets of landscape metrics selected for the genetic linker (Step 1 Models). Statistically significant effects (*p* < .05) are represented by solid lines with 95% confidence bands depicted in grey. Partial residuals are represented by filled squares. Only models with at least one significant term are shown.

In total, we fitted 10 Step 2 Models (Table 3), categorized into two sets of landscape metrics for each population genetic measure of *P. multiflorum*. We found significant effects of area‐based landscape metrics, which had been selected to explain the pollinator movement indicators, at 2000 m buffer sizes. Maize cover had a positive effect on *A*_*r*_ (Model 2a, Figure 3a) and *F* (Model 2g, Figure 3j), but a negative effect on *H*_*o*_ (Model 2e, Figure 3f) of *P. multiflorum* (Table 3). In contrast, the cover of semi‐natural grassland had a negative effect on *A*_*r*_ (Model 2b, Figure 3c) and *F* (Model 2h, Figure 3l), but a positive effect on *H*_*o*_ (Model 2f, Figure 3h). Additionally, index variables had significant effects at smaller buffer distances within the same models. For instance, landscape heterogeneity within a buffer distance of 125 m positively affected *A*_*r*_ (Model 2a, Figure 3b), while the edge density within a 250 m buffer exhibited an unimodal effect on *H*_*o*_ with a maximum above the mean edge density (Model 2f, Figure 3i). For *H*_*e*_ and *PolD*_PS_, no significant landscape effects could be detected in Step 2 Models.

**TABLE 3:** Summary of 10 Step 2 Models describing landscape effects on the population genetic structure of *Polygonatum multiflorum*.

Step 2 Model	Step 1 Model compared	Population genetic measure	Included landscape metrics with regression coefficient and *p*‐value			*r* ^*2*^	∆AICc
Model 2a	Model 1a/1c	*A*_*r*_	MAIZE2000	LANDHET125		.33/.33	13.83
			*b* = 0.50, *p* = .0445	*b* = 0.52, *p* = .0385			
Model 2b	Model 1b	*A*_*r*_	**SEMNATGRASS2000**	**EDGEDEN250**	**EDGEDEN250** ^**2**^	.30/.30	19.17
			*b* = −0.56, *p* = .0329	*b* = 0.34, *p* = .1694	*b* = −0.02, *p* = .9162		
Model 2c	Model 1a/1c	*H*_*e*_	**MAIZE2000**	**LANDHET125**		.04/.14	13.78
			*b* = 0.21, *p* = .5285	*b* = 0.1, *p* = .7271			
Model 2d	Model 1b	*H*_*e*_	**SEMNATGRASS2000**	**EDGEDEN250**	**EDGEDEN250** ^**2**^	.2/.2	15.64
			*b* = −0.26, *p* = .3135	*b* = 0.27, *p* = .3009	*b* = −0.3, *p* = .1733		
Model 2e	Model 1a/1c	*H*_*o*_	**MAIZE2000**	**LANDHET125**		.41/.66	11.31
			*b* = −0.80, *p* = .0469	*b* = −0.24, *p* = .2401			
Model 2f	Model 1b	*H*_*o*_	**SEMNATGRASS2000**	**EDGEDEN250**	**EDGEDEN250** ^**2**^	.72/.72	2.82
			*b* = 0.61, *p* = .0012	*b* = 0.36, *p* = .0263	*b* = −0.36, *p* = .0106		
Model 2 g	Model 1a/1c	*F*	**MAIZE2000**	**LANDHET125**		.63/.63	9.02
			*b* = 0.86, *p* = .0002	*b* = 0.25, *p* = .1564			
Model 2 h	Model 1b	*F*	**SEMNATGRASS2000**	**EDGEDEN250**	**EDGEDEN250** ^**2**^	.64/.64	12.95
			*b* = −0.77, *p* = .005	*b* = −0.14, *p* = .3915	*b* = −0.14, *p* = .3280		
Model 2i	Model 1d/1e	*D*_PS_	**SEMNATGRASS1to3**	**MAIZE1to7**	**MAIZE1to7** ^**2**^	.02/.35	26.77
			*b* = −0.11, *p* = .6795	*b* = 0.10, *p* = .6312	*b* = −0.07, *p* = .6875		
Model 2j	Model 1d/1e	*D*_PS_	**SEMNATGRASS1to3**	**MAIZE1to7**		.01/.36	28.1
			*b* = −0.13, *p* = .6282	*b* = −0.14, *p =* .4887			

*Note*: At the node level, allelic richness (*A*_*r*_), expected (*H*_*e*_) and observed heterozygosity (*H*_*o*_) and the *F*‐value are used as response variables and at the link level *PolD*_PS_. Presented are the included landscape metrics for each model, the marginal/conditional *r* ^*2*^ values and the ∆AICc in comparison to the model with the lowest AICc among Step 3 Models best for the respective population genetic measure.

### 3.3 Comparison of Step 2 Models with Step 3 Models

**FIGURE 4:**
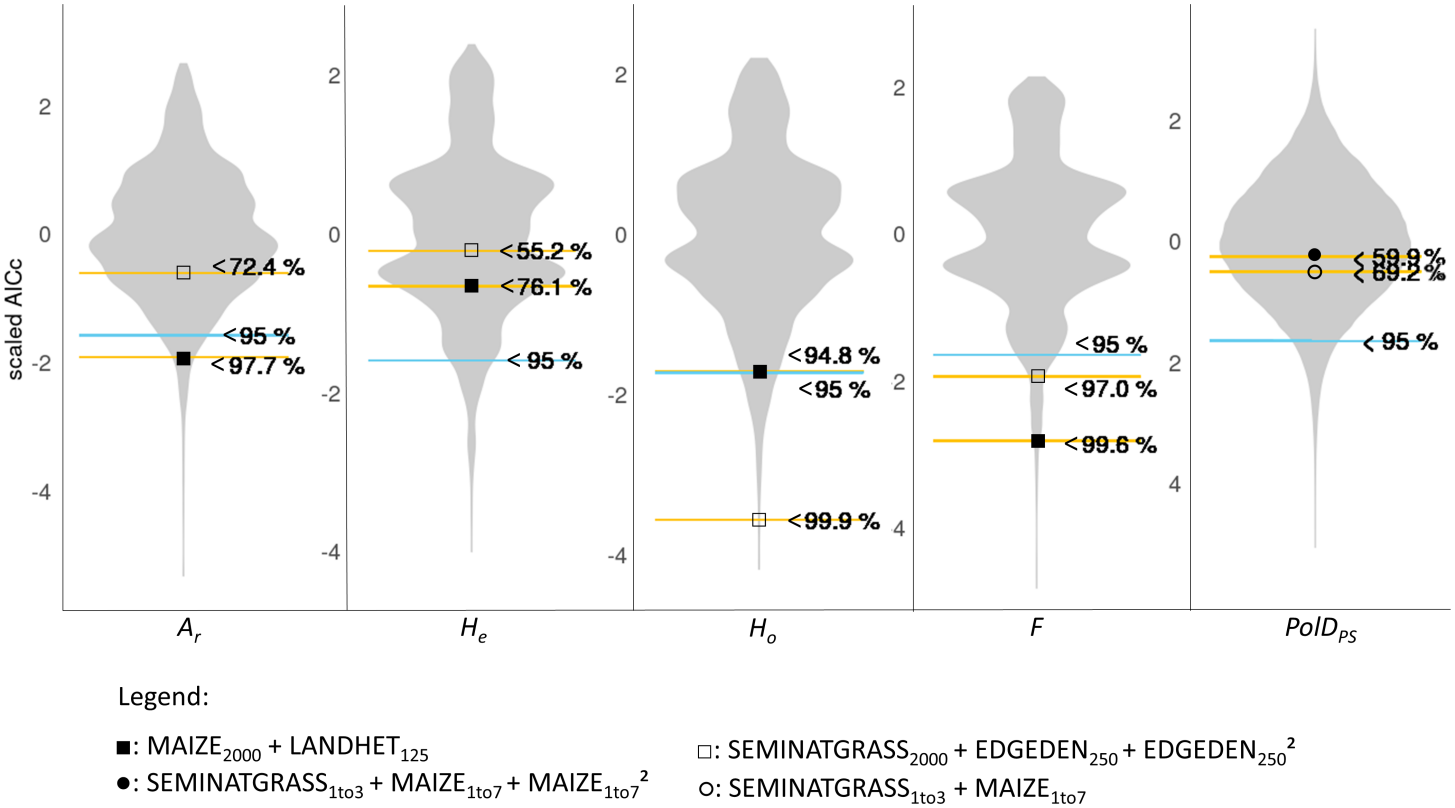
Visualization of the model comparison between Step 2 Models and Step 3 Models (cf. Table S7). The grey kernel density plot illustrates the distribution of AICc values of all Step 3 Models, considering the five population genetic measures of *Polygonatum multiflorum*, that is, allelic richness (*A*_*r*_), expected (*H*_*e*_) and observed heterozygosity (*H*_*o*_), the *F*‐value (*F*) at the node level and *PolD*_PS_ at the link level. For each measure, the yellow lines depict AICc values of Step 2 Models within the distribution of Step 3 Models, while the blue lines indicate the 95% boundary. The symbols indicate the sets of landscape metrics. AICc values were scaled.

According to AICc values, four Step 2 Models ranked lower than 95% of Step 3 Models for the specific population genetic measure (Figure 4, Table S7). These four models are interpreted as performing better than expected by chance. Specifically, Model 2a ranked lower for *A*_*r*_, Model 2f for *H*_*o*_ and Models 2g and 2h for *F*. Model 2e ranked lower than 94.8% of Step 3 Models for *H*_*o*_ and, thus, was only slightly under the threshold of 95%. For *H*_*e*_ and *PolD*_PS_, Step 2 Models performed at best better than 76% and 69% of Step 3 Models respectively.

Three to five models were identified as best models with ∆AICc < 2 (Step 3 Models best) for the different genetic diversity measures (*A*_*r*_, *H*_*e*_, *H*_*o*_, *F*) of *P. multiflorum* (Table 4, Table S8.1, Figure S8.2). In contrast, for *PolD*_PS_, there were 55 models below ∆AICc < 2. The landscape metrics, which occurred most frequently in Step 3 Models best, were LANDHET for *A*_*r*_, SEMNATVEG for *H*_*e*_, MAIZE for *H*_*o*_, SEMNATGRASS and C‐FOREST for *F* and L_ROAD for *PolD*_PS_. Three of six of these were also found in Step 2 Models. Additionally, some of the landscape metrics in Step 3 Models best shared identical buffer sizes with Step 2 Models, such as MAIZE2000 for *H*_*e*_, *H*_*o*_ and *F*, as well as SEMINATGRASS2000 for *F*. However, none of the Step 3 Models best shared more than one term with Step 2 Models. Certain landscape metrics like SEMNATVEG, CEREALS, RAPESEED, GRASS, ORCHARD, SETTLE, L_ROAD, L_FRINGE, C_FOREST and D_FOREST were absent in any Step 2 Model, but appeared in Step 3 Models best.

**TABLE 4:** Summary of Step 3 Models best.

Measure	*N* Step 3 Models best	Landscape metrics	Buffers/width‐to‐length ratio
*A*_*r*_	3	LANDHET* (3)	250, 500
		D_FOREST (2)	125
		GRASS (1)	2000
*He*	5	SEMNATVEG (4)	2000
		MAIZE* (3)	2000*, 1000, 500, 250
		WATER (1)	2000
		ORCHARD (1)	1000
*H*_*o*_	5	MAIZE* (5)	2000*, 1000
		D_FOREST (2)	500
		C_FOREST (2)	500, 250
		L_FRINGE (1)	250
*F*	3	SEMNATGRASS* (2)	2000*
		C_FOREST (2)	250, 125
		MAIZE* (1)	2000*
		L_FRINGE (1)	500
*D*_PS_	55	L_ROAD (54)	1 to 2, 1 to 3, 1 to 2
		ORCHARD (34)	1 to 2, 1 to 3, 2 to 3
		L_WATER (19)	1 to 2, 1 to 3, 1 to 5, 2 to 3
		RAPESEED (18)	1 to 2, 1 to 3, 2 to 3
		CEREAL (17)	1 to 2, 2 to 3
		MAIZE* (17)	1 to 2, 1 to 3, 2 to 3
		EDGEDEN (17)	1 to 2, 1 to 3, 2 to 3
		GRASS (10)	1 to 2, 1 to 5, 2 to 3
		LANDHET (10)	1 to 7, 2 to 3
		L_WOOD (10)	1 to 2, 1 to 3, 1 to 7, 2 to 3
		D_FOREST (5)	1 to 2, 2 to 3
		SEMNATVEG (4)	1 to 3, 2 to 3
		SEMNATGRASS* (2)	1 to 5, 2 to 3
		SETTLE (1)	2 to 3

*Note*: The table displays the number of Step 3 Models best with ∆AICc < 2, the count of corresponding landscape metrics in these models and the corresponding landscape buffers or width‐to‐length ratios involved. Landscape metrics marked with an asterisk occurred also in Step 2 Models.

## 4 DISCUSSION

With our integrated approach, we showed that the landscape composition significantly influenced the movement of *B. pascuorum* among isolated populations of the forest herb *P. multiflorum*, which confirmed our first hypothesis. We also showed for *A*_*r*_, *H*_*o*_ and *F* that landscape effects on the pollinator movement activity can be translated into landscape effects on the forest herb's population genetic structure. These results partially confirmed our second hypothesis.

### 4.1 The movement activity of the genetic linker is sensitive to landscape effects at different spatial scales

Our results demonstrated that the mechanisms behind the landscape effects on the movement activity of *B. pascuorum* cannot solely be derived from studies on its abundance. Such studies aimed to predict the abundance of *B. pascuorum* from the landscape composition at different buffer sizes and identified the best models for predicting visitation rates and abundances at buffer sizes of 1000 m (Knight et al., 2009; Westphal et al., 2006). They showed that land‐use types with high floral resources, such as rapeseed fields, were most relevant to explain the numbers of flower visitors and nests because they promoted colony development and survival. However, whether workers of *B. pascuorum* move among‐forest patches does not necessarily depend on colony numbers or sizes.

Our study, focusing on movement indicators, revealed two distinct scales of influence: a local scale with buffer sizes of 125 or 250 m and a landscape scale with buffer sizes of 2000 m (Table 2, Figure 2). Effects of landscape composition on the foraging behaviour of bumblebees across multiple spatial scales have been demonstrated before (Jha & Kremen, 2013). At the landscape scale, we propose that our findings reflect the relative attractiveness of the forest herb's floral resources in the specific landscape context, while at the local scale, the mechanisms behind our results might involve resource complementation, nest distribution or bumblebees' navigation patterns. In the following, we will explore the mechanisms at both scales.

### 4.2 Context‐specific value of the forest herb's floral resources on the landscape scale

The *Circe principle* describes landscapes as mosaics of habitats, in which the relative resource value of each habitat depends on the resources provided by the neighbouring habitat types (Lander et al., 2011). This aligns with studies showing that bumblebees' foraging decisions depend on the relative value of habitat within a specific spatial and temporal context (Bontšutšnaja et al., 2021; Jha & Kremen, 2013; Proesmans et al., 2019). Applied to our results that *NESTS*_*shared*_ and *FOREST‐PATCHES*_*shared*_ increased with MAIZE_2000_ (Model 1a, Model 1c) and decreased with SEMNATGRASS_2000_ (Model 1b), this rationale suggests that the contrasting features of semi‐natural grassland and intensively managed maize fields influence the value of forest as a foraging habitat (Jakobsson & Ågren, 2014). Such opposing effects of (semi‐natural) grassland and maize on the total bumblebee abundance in wheat fields have been demonstrated at buffer sizes of 1000 m (Alignier et al., 2023).

Semi‐natural grassland is often considered among the most attractive foraging and nesting habitats for insects in agricultural landscapes (Ekroos et al., 2013, 2015), which is also true for *B. pascuorum* (Goulson et al., 2010). It offers abundant and diverse floral resources (Johansen et al., 2022), also during the flowering period of *P. multiflorum* (Jachuła et al., 2022). In contrast, maize fields are known to provide hardly any floral resources, even less than other wind‐pollinated crops because of high inputs of fertilizers and herbicides which minimize the abundance of any wildflowers (Alignier et al., 2023; Fagúndez et al., 2016; Hass et al., 2019; Kleijn & Verbeek, 2000). Furthermore, because maize fields remain bare soil during the flowering period of *P. multiflorum* in our region (Figure S1D), we consider them as highly unattractive for pollinators.

Following the Circe principle, the difference in the relative values among potential foraging habitats leads to a higher frequency of traversing resource‐poor land‐use types by the genetic linkers. While our node‐level results align with this logic, our link‐level results contradict this interpretation. *BomD*_PS_ increased both with higher MAIZE_1to7_ and with SEMNATGRASS_1to3_ (Models 1d and 1e), suggesting that workers avoid flying over maize fields. Combining both levels suggests that, in our case, avoiding directions with high maize cover prompts genetic linkers to fly more directly towards forest, enhancing the relative value of nearby forests as foraging habitat. In contrast, semi‐natural grassland likely affected *BomD*_PS_ by attracting the workers through the provision of plenty of floral resources, thereby reducing the relative value of forest as foraging habitat, which is consistent with our findings at the node level. If these interpretations hold true, they exemplify how utilizing both levels allows us to uncover a counterintuitive way, by which barriers can also enhance gene flow (Storfer et al., 2010), that is, redirect movement along the barrier.

### 4.3 Mechanisms of how landscape heterogeneity increases among‐forest patch movement activity at the local scale

At the local scale, the landscape heterogeneity was positively correlated with *NESTS*_*shared*_ and *FOREST‐PATCHES*_*shared*_ (Models 1a and 1c, Figure 2, Table 2), and edge density (Model 1b, Figure 2, Table 2) had a marginal significant quadratic effect on *NESTS*_*shared*_. These findings elucidate how the surrounding landscape influences the extent to which forest patches share foraging bumblebees from the same nests. There are three non‐exclusive explanations for this pattern:

1. The first explanation is given by landscape complementation (Ammann et al., 2024; Clake et al., 2022; Fahrig et al., 2011). Diverse habitats in a smaller area may provide more foraging resources (Pywell et al., 2006; Rundlöf et al., 2008). Additionally, the boundary structures among different land‐use patches can also provide floral resources (Happe et al., 2018). As a consequence, foraging workers might prefer moving among clusters of complementary land‐use types rather than among isolated single‐habitat spots if those clusters provide more (diverse) floral resources (Jha & Kremen, 2013). In a previous study by Feigs et al. (2022), they showed that, within the same landscape windows, the mean distances between forest patches that shared *B. pascuorum nests* was 2.4 km, indicating that at least a part of the observed bumblebee workers moved among multiple clusters of complementary land‐use types even over longer distances.
2. More diverse landscapes, habitat boundaries and linear features provide more suitable nesting sites for bumblebees (Kells & Goulson, 2003; Meyer et al., 2005; Osborne et al., 2008; Svensson et al., 2000). This could lead to a higher number of nests near the forest patch, resulting in frequent entries and exits. If bumblebee nests are instead within the forest habitat, workers might rarely visit other forest patches during spring if sufficient flower resources exist within the patch to sustain the nests.
3. Bumblebees exhibit complex navigation abilities (Brebner et al., 2021; Fragoso et al., 2021; Osborne et al., 2013), with landmarks likely being one of the crucial components. Within more complex landscapes, bumblebees appear to navigate more effectively (Cranmer et al., 2012; Plowright & Galen, 1985; Van Geert et al., 2010). An increased diversity of surrounding landscape features might enhance the chances for forest patches to be recognized and relocated (Hass et al., 2019). If bumblebees are guided to or better remember patches, it could elevate the likelihood of revisiting these specific forest patches multiple times.

### 4.4 Landscape effects on genetic linkers can be translated into landscape effects on the forest herb's population genetic structure

Two primary observations suggest that landscape effects on the genetic linker translate into landscape effects on the population genetics of the forest herb. First, for *A*_*r*_, *H*_*o*_ and *F*, the Step 2 Models met the criterion of performing better than 95% of Step 3 Models. Secondly, for the same measures, several of the terms suggested in Step 1 were found to be significant in Step 2 as well. These observations are noteworthy, considering the long generation time of the clonal and long‐lived forest herb species, which can span many decades (Kosiński, 2015). We anticipated time delays between any landscape change, the resulting shifts in pollinator movement behaviour and subsequent changes in the population genetic structure of forest herbs. This delay occurs because bumblebees have shorter generation times (one generation per year) than perennial plants, and because forest herbs respond to movement pattern of the genetic linkers and not directly to the landscape (Liu et al., 2015).

As described above, in landscapes with fewer floral resources, the value of forest herbs' pollen and nectar for the genetic linkers should increase, leading to more pollen‐driven gene flow among *P. multiflorum* populations. At the landscape scale, we found that the allelic richness of *P. multiflorum* populations increased with MAIZE_2000_ (Model 2a), whereas it decreased with SEMINATGRASS_2000_ (Model 2b). These effect directions are interpretable in a straightforward manner. In contrast, assessing the effects on *H*_*o*_ and *F* requires considering that 15 of 17 populations of *P. multiflorum* displayed heterozygote excess, resulting in significantly negative *F*‐values (Feigs et al., 2022; Naaf et al., 2021). This excess stems likely from a large proportion of clonal reproduction (Reichel et al., 2016) and the dominance of a few pollen donors within populations in the past (Pudovkin et al., 1996; Stoeckel et al., 2006). We found that MAIZE_2000_ positively affected the genetic linker movement activity, that is, increased *F* (Model 2g) and decreased *H*_*o*_ (Model 2e). Conversely, a higher SEMINATGRASS_2000_ decreased *F* (Model 2h) and increased *H*_*o*_ (Model 2f). This means that in landscapes with a higher dominance of maize, forest herb populations tend to reach Hardy–Weinberg equilibrium (a balance between genes and genotypes, comparable to a situation where all parents contribute equally to the populations' genotypes (Crow, 2001)) faster, which becomes evident in *F*‐values closer to 0 and lower *H*_*o*_ values due to increased pollen‐mediated gene flow. In contrast, in landscapes with more semi‐natural grassland, the genetic linker exhibits reduced movement among‐forest patches, resulting in less pollen‐mediated gene flow among populations of *P. multiflorum*.

The strong impact of maize cultivation on the genetic linker, and consequently, on forest herb populations, is remarkable, especially considering the anticipated time delay in response to the herb's population genetic structure and the short history of maize within these landscapes of at most 60 years (von Redwitz & Gerowitt, 2018). Our sampling covered a high variance of different levels of maize dominance. We have to acknowledge that this variance was nested according to our landscape windows (Figures 2 and 3). It is possible that the observed effects of maize and semi‐natural grassland could just mirror other differences between these landscape windows, such as unmeasured landscape features or any characteristics of the linker, the forest herb or the forest patches. Additionally, differences in climatic conditions across these landscape windows cannot be ruled out. Still, we are convinced that the observed pattern is due to the stark differences in MAIZE_2000_ and SEMINATGRASS_2000_ among the landscape windows (S2 and S3). This is supported by our results at the link level, at which maize cover was less nested within landscape windows (Figure 2g,i). To further explore our findings, a subsequent study should be conducted in landscape windows with a stronger gradient from low to high maize and semi‐natural grassland cover within a landscape, or a higher replication over more than three landscapes.

### 4.5 The limits of translating landscape effects on genetic linkers to those on forest herbs

Our integrated approach also revealed the limits for translating the landscape effects from bumblebee movement indicators to the herb's population genetic measures. The limits became evident in that none of the sets of landscape metrics detected in Step 1 had been selected as optimal to explain the plant population genetic measures in Step 3. This emphasizes the need for caution when attributing patterns in a plant's population genetic structure to the expected behaviour of its most likely genetic linker (Kramer et al., 2011; Lanes et al., 2018; Stoll et al., 2020). The limits can be found both on the side of the movement indicators as well as on the side of the population genetic measures.

On the side of the movement indicators, landscape effects need to be relatively strong and stable over time to become evident. Otherwise, as we observed with the relatively low marginal *r* ^*2*^ for *BomD*_PS_ (<.1; Models 1d, e), it is unlikely that such effects will be traceable in the plant's population genetic structure (Models 2i, j). Also, landscape elements might affect the genetic linkers in multiple ways at the same time. If it holds true that a higher landscape heterogeneity at the local scale increases the movement activity because of the higher floral resources provided by complements including forest patches and other habitat types as elaborated above, this would also mean that workers collect pollen from a larger variety of plants. Consequently, flower constancy would decrease, which is one of the main factors determining chances of successful pollen transport (Popic et al., 2013). This could be an explanation for why we did not find significant effects of LANDHET_125_ on *H*_*o*_ and *F* in the Step 2 Models (Figure 3).

Another reason for the lack of effects of land use metrics at the local scale in some of the Step 2 Models could be that a more diverse landscape surrounding a forest patch might also enhance the local pollination service provided by *B. pascuorum* individuals within the forest patch (Ekroos et al., 2015). This would lead to a higher reproduction rate dominated by those large clones within the forest patches (see previous subsection). This second argumentation better elucidates why we found significant effects in Step 2 Models for *A*_*r*_ (Model 2a), which is more sensitive to the new introduction of alleles, but not for *H*_*o*_ (Model 2e) and *F* (Model 2g), which rather reflect the equilibrium of the allele composition within a population (Greenbaum et al., 2014).

On the side of the forest herb, the population genetic measures reflect an accumulation of different effects over many years. This might explain why the Step 3 Models best included landscape metrics that could not be linked to the recent movement activity of *B. pascuorum*. Even though Step 2 Models exhibited quite large marginal and conditional *r* ^2^‐values in some cases, only for *H*_*o*_, the *r* ^2^‐values of Step 2 Models and Step 3 Model best were at a comparable level (Model 2f: .72/.72; Model 3i: .76/.76). At least three different mechanisms that cover multiple years could be reflected in the Step 3 Models best that are beyond what Step 2 Models could capture:

First, the agricultural landscape is under constant change. Other landscape elements might have affected the movement activity of *B. pascuorum* in the past but might be of lower importance in the present, for instance, due to shifts in dominant crop types at the landscape level. Therefore, their effects are not traceable in the movement indicators anymore but still in the population genetic structure of the forest herb. This might be true for the effects of cereals or rapeseed on *PolD*_PS_ (Table 4, Table S8.1) or the semi‐natural vegetation for *H*_*e*_ (Models 3 e‐h, Table 4, Table S8.1).

Second, alternations in landscape composition are also known to result in shifts in the pollinator communities (Vray et al., 2019). Notably, the proportion of grassland holds a recognized influence on the composition of bumblebee species (Vray et al., 2019). Such shifts in the pollinator communities can also include changes in the main pollinator species. For instance, if landscapes once had a higher forest cover, the main pollinator of *P. multiflorum* could have been a bumblebee species that prefers forest as habitat, such as *Bombus hypnorum* (Crowther et al., 2014). The transition of more habitat‐specialized pollinators to less habitat‐specialized pollinators like *B. pascuorum* is an anticipated consequence of landscape fragmentation (Hadley & Betts, 2012). Correspondingly, the forest herb's population genetic structure might still bear the imprint of higher forest cover present in the studied landscape only a few hundred years ago (Huang et al., 2024).

Third, even though the importance of long‐distance seed dispersal vectors for *P. multiflorum* is unknown, the fleshy berries imply that seed vectors such as forest birds or mammals might also contribute to gene flow of *P. multiflorum* (Johnson et al., 1985). If these seed vectors are forest species, they should also respond to forest cover (Heikkinen et al., 2004; Radford & Bennett, 2007). Even if seed dispersal events are rare, they should manifest themselves in the plant's population genetic structure over many years.

A major limitation of our integrated approach was that it could only explain the specific part of the population genetic structure of *P. multiflorum* that correlated with landscape metrics influencing the movement activity of *B. pascuorum*. This leaves a large part of landscape effects on the overall population genetic structure of *P. multiflorum* unaddressed. However, our findings suggest expanding our approach to include movement indicators of multiple important genetic linker species. Such an analysis would allow us to differentiate which landscape metrics influence the activity of one or multiple genetic linker species as well as the specific effect strengths and directions. Theoretically, it could also include (multiple) seed vectors, which in the case of *P. multiflorum* would have to be identified first.

Future research on the effects of landscape composition should also more directly address the temporal scales, which are reflected by the forest herbs' population genetic structure and the genetic linkers' movement indicators (Balkenhol et al., 2009). Regarding the past, this means including historical landscape metrics from multiple points in time to determine how long the patterns, displayed by current population genetic measures as well as by the linkers' movement indicators, date back. Concerning the present, paternity analysis of the forest herb populations would allow to quantify the amount of contemporary pollen flow among populations per year (Holderegger et al., 2010) and would provide insights into how this pollen flow is related to effects of the current landscape composition on the contemporary linker movement activity.

## 5 CONCLUSION

Our research uncovered that the among‐forest patch movement activity of *B. pascuorum* is influenced by the landscape composition both at the landscape and the local scale. The mechanisms we employ to interpret these effects cannot be directly inferred from knowledge about landscape effects on abundances of *B. pascuorum*. This underscores the importance of separately examining each ecological function of an organism. In the concrete case of the pollinator *B. pascuorum*, this necessitates careful distinction between the ways in which the landscape affects its function for forest herbs' seed set and recruitment and its function as their genetic linker.

Our study further demonstrated the feasibility of translating landscape effects on the movement activity of a genetic linker into landscape effects on the population genetic structure of a plant. Notably, this was also possible for landscape elements relatively recently introduced, such as maize. Consequently, the recent activity of the genetic linker is responsible for a considerable proportion of individuals in the forest herb populations, which are thus relatively young and of sexual origin. This observation is noteworthy, as it demonstrates that not only processes over centuries but also those occurring within a few decades, such as shifts in crop type dominance, contribute to shaping the evolutionary potential of this long‐living and clonal forest herb. In conclusion, our findings underscore the importance of integrating the distribution of floral resources on a landscape scale into conservation approaches aiming at increasing the functional connectivity of long‐living species such as clonal forest herbs.

## AUTHOR CONTRIBUTIONS

**Jannis Till Feigs:** Conceptualization (lead); data curation (lead); formal analysis (lead); investigation (lead); methodology (lead); resources (lead); software (lead); visualization (lead); writing – original draft (lead); writing – review and editing (lead). **Siyu Huang:** Visualization (supporting); writing – original draft (supporting); writing – review and editing (supporting). **Jörg Brunet:** Supervision (supporting); writing – original draft (supporting). **Martin Diekmann:** Supervision (supporting); writing – original draft (supporting). **Per‐Ola Hedwall:** Writing – original draft (supporting). **Katja Kramp:** Funding acquisition (equal); methodology (supporting); project administration (supporting); resources (equal); software (supporting); supervision (supporting). **Stephanie I. J. Holzhauer:** Conceptualization (supporting); funding acquisition (equal); methodology (supporting); project administration (supporting); supervision (supporting); writing – original draft (supporting). **Tobias Naaf:** Conceptualization (supporting); data curation (supporting); formal analysis (supporting); funding acquisition (equal); investigation (supporting); methodology (supporting); project administration (lead); resources (equal); software (equal); supervision (lead); validation (supporting); visualization (supporting); writing – original draft (supporting); writing – review and editing (supporting).

## FUNDING INFORMATION

We thank the German Research Foundation as funders of this research (research grants NA 1067/2–1, HO 4742/2–1 and KR 5060/1–1). This includes the research work of JTF, KK, SH, SIJH and TN. This work was supported by the FWO Scientific research network FLEUR (http://www.fleur.ugent.be). This research was also partly funded by the German Federal Ministry of Food and Agriculture (BMEL) and the Ministry for Science, Research and Culture of the State of Brandenburg (MWFK).

## CONFLICT OF INTEREST STATEMENT

The authors declare that the research was conducted in the absence of any commercial or financial relationships that could be construed as a potential conflict of interest.

## Supporting information


Data S1.

## Data Availability

All datasets are accessible on DRYAD: The node‐level data for *P. multiflorum* via https://doi.org/10.5061/dryad.tb2rbp00k, the link‐level data for *P. multiflorum* via https://doi.org/10.5061/dryad.h70rxwdkf, the allele tables of the pollinators via https://datadryad.org/stash/dataset/10.5061/dryad.sf7m0cg8w and all landscape metrics via https://doi.org/10.5061/dryad.h70rxwdkf. The R‐script for the analysis is stored as supplementary files.
