# Supplementary material for: Bumblebees mediate landscape effects on a forest herb's population genetic structure in European agricultural landscapes
Source: Ecol Evol. 2024 Jul 25;14(7):e70078. doi: 10.1002/ece3.70078 (PMC11269766; doi:10.1002/ece3.70078)
Supplement: Supplementary file 1 — Data S1. [file ECE3-14-e70078-s001.zip › Forest_herb_Bunblebee_Landscape_Supplement.pdf]

Supplementary material

Bumblebees mediate landscape effects on a forest herb's population genetic structure in European agricultural landscapes

Table of contents

S1 Photos from field work 2

S2 Landscape composition maps 3

S3 Bumblebee movement indicators 10

S4 Measures of forest herb's genetic structure and linker's movement activity 11

S5 Univariate models 13

S6 Tests for collinearity 14

S7 Comparison Step 2 Models and Step 3 Models 16

S8 Step 3 Models best 17

S1 Photos from field work

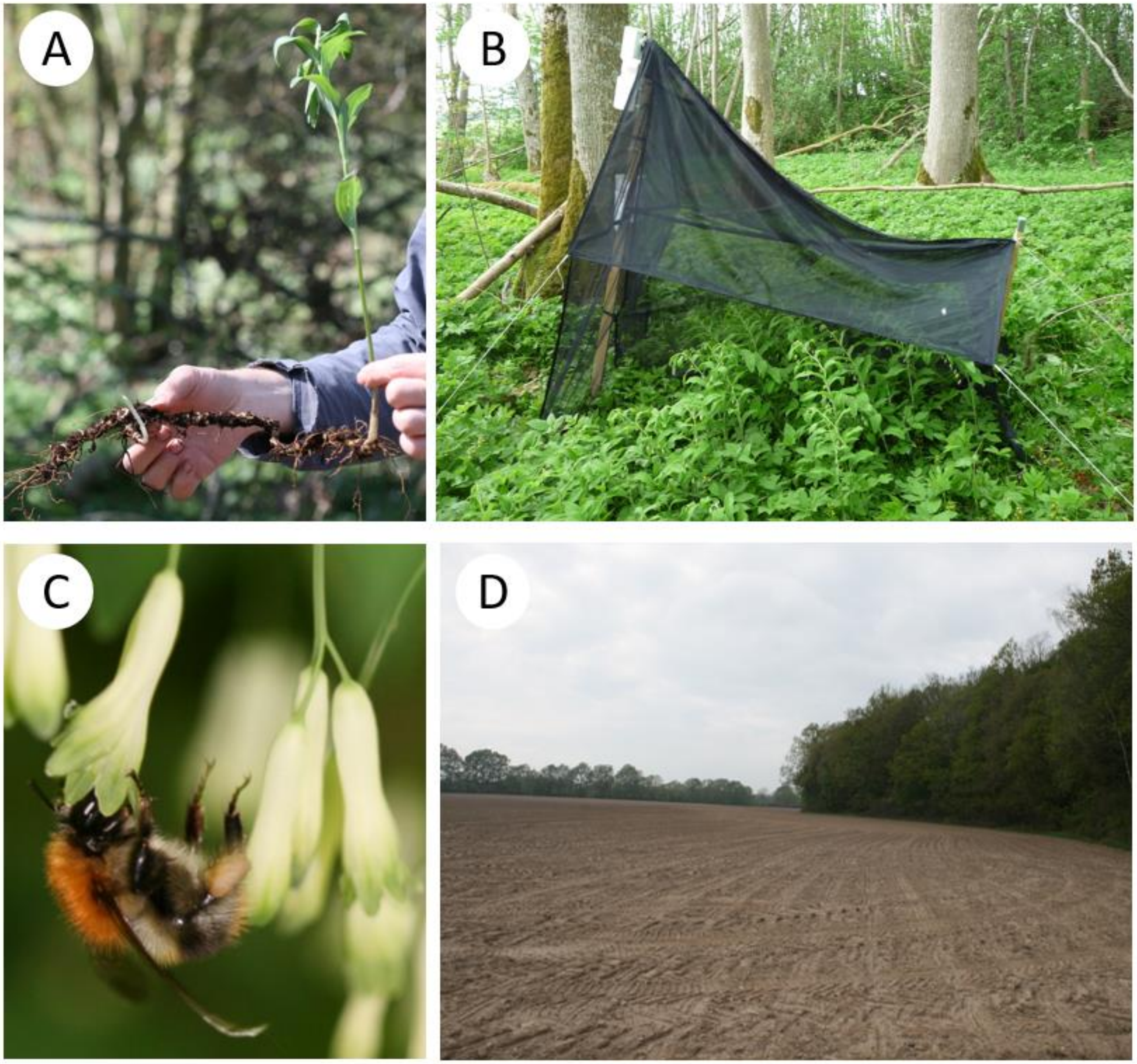

Figure S1: Photos taken during field work in 2018 and 2019: A) Depicts a specimen of *Polygonatum multiflorum*, displaying its leaves, stem and a new clonal shoot; B) Illustrates a typical arrangement of a Malaise trap within a flowering patch of *P. multiflorum* individuals; C) Displays a *Bombus pascuorum* individual visiting flowers of *P. multiflorum* in a forest patch; D) Shows the state of a maize field in the eastern German landscape window during the blossom of *P. multiflorum* (May 2nd, 2019).

S2 Landscape composition maps

S2.1 Area based

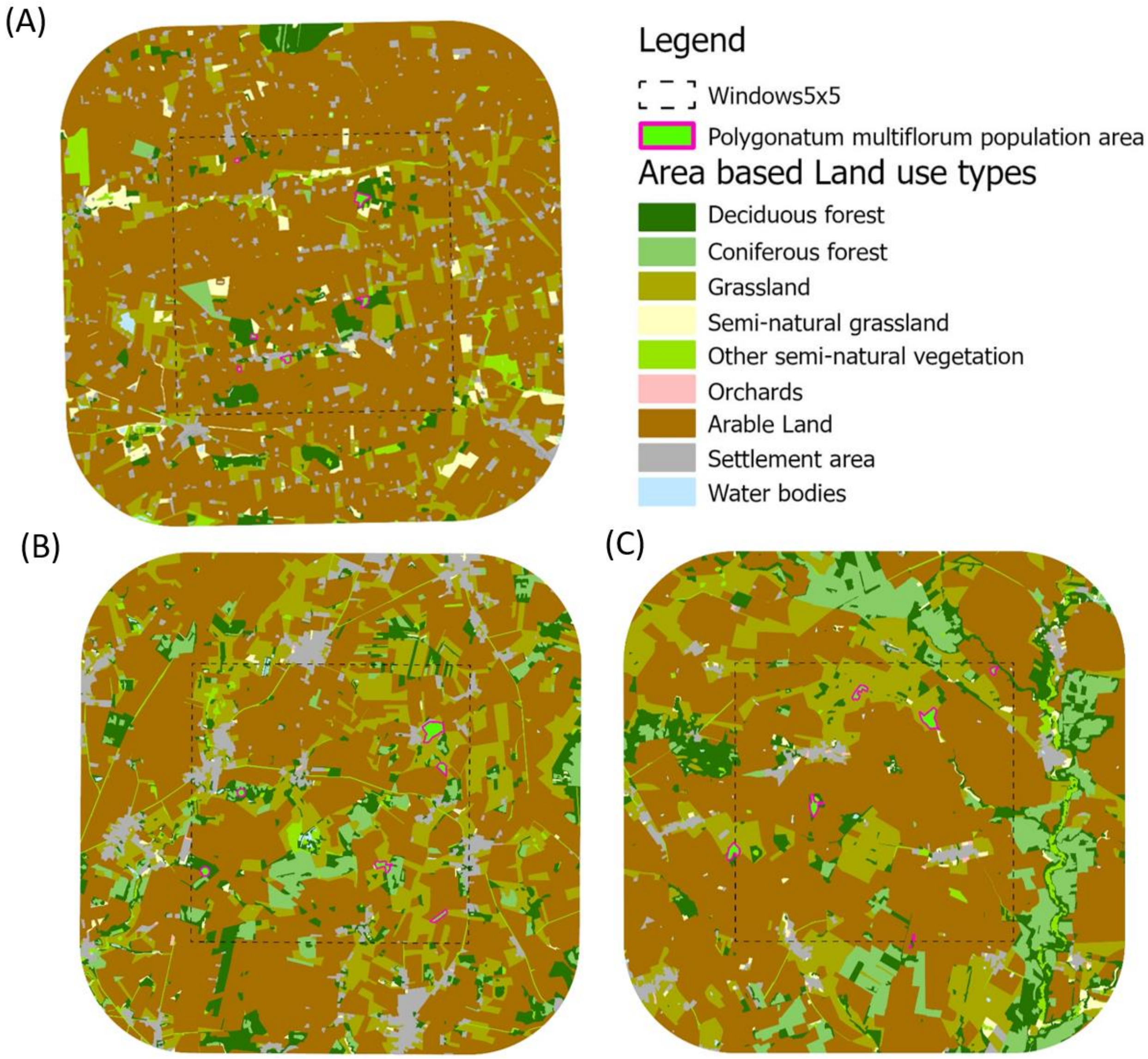

Figure S2.1: 7 km x 7 km maps around the 5 km x 5 km landscape windows in (A) Southern Sweden, (B) Eastern Germany, and (C) Western Germany. The maps show the distribution of area based land-use types and the 5 km x 5 km landscape windows in which samples where taken.

S2.2 Linear landscape elements

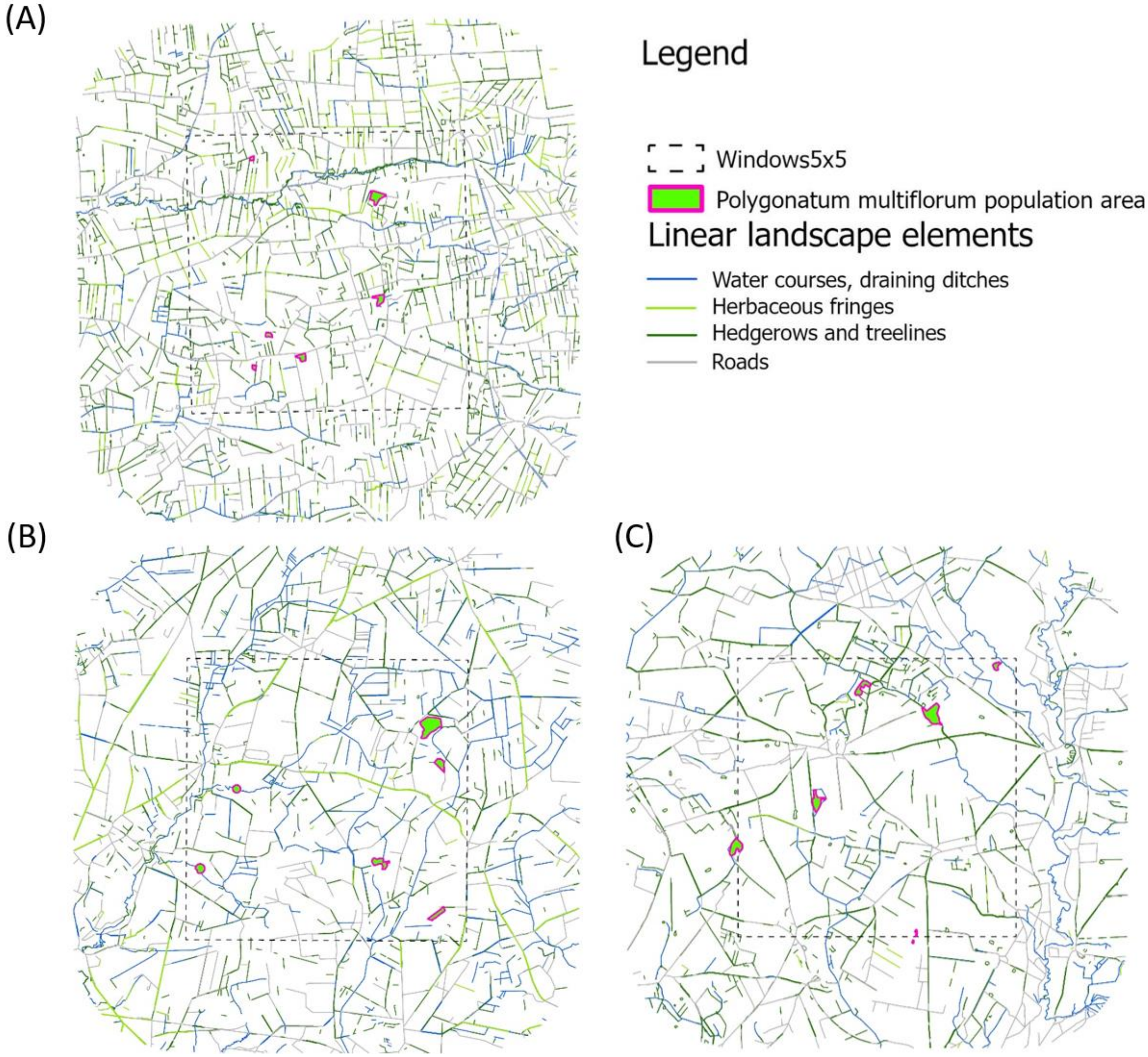

Figure S2.2: 7 km x 7 km maps around the 5 km x 5 km landscape windows in (A) Southern Sweden, (B) Eastern Germany, and (C) Western Germany. The maps show the distribution of linear landscape elements.

S2.3 Cereal cover

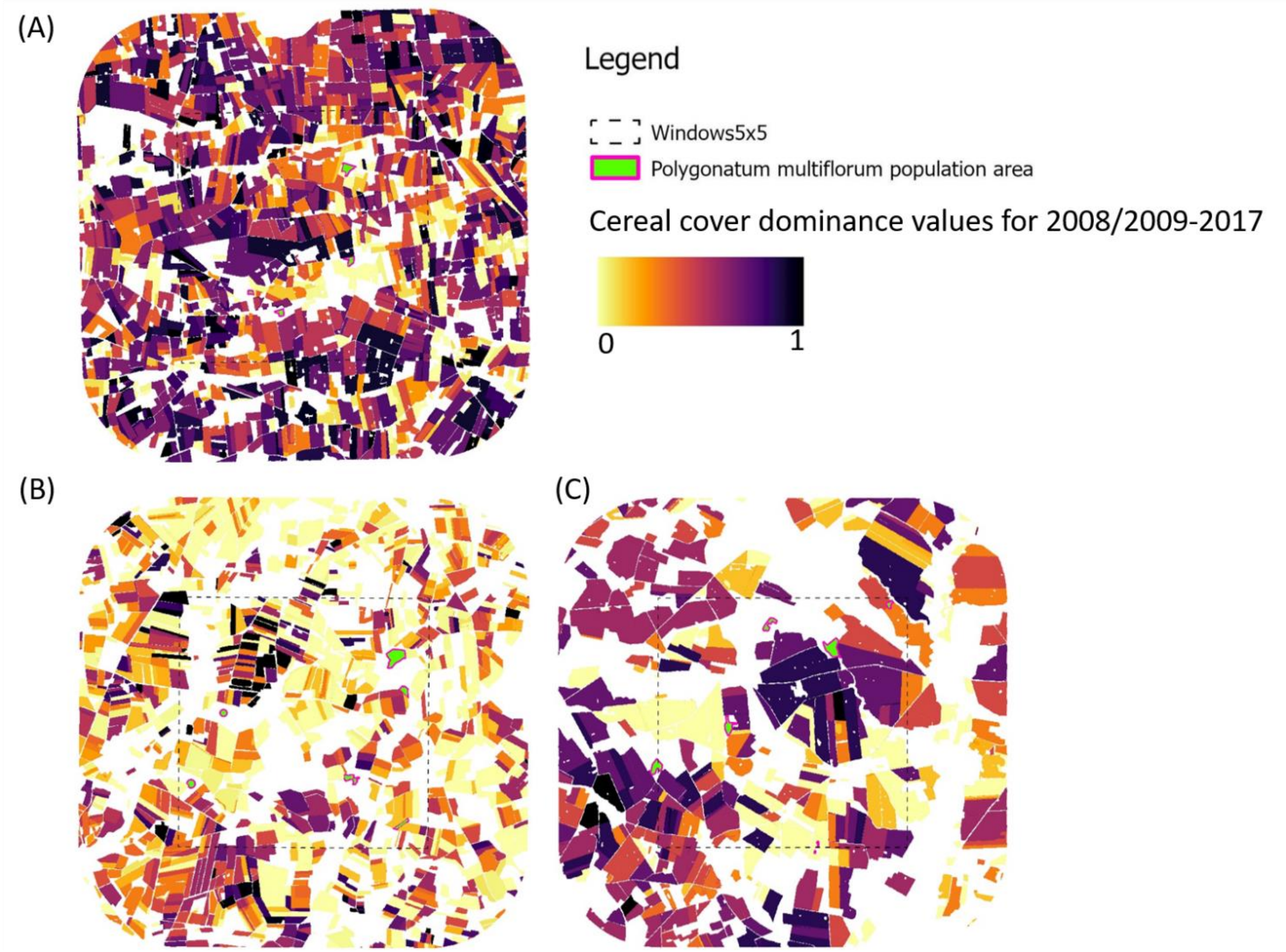

Figure S2.3: 7 km x 7 km maps around the 5 km x 5 km landscape windows in (A) Southern Sweden, (B) Eastern Germany, and (C) Western Germany. The maps show the degree of cereal cover dominance per polygon.

S2.4 Maize cover

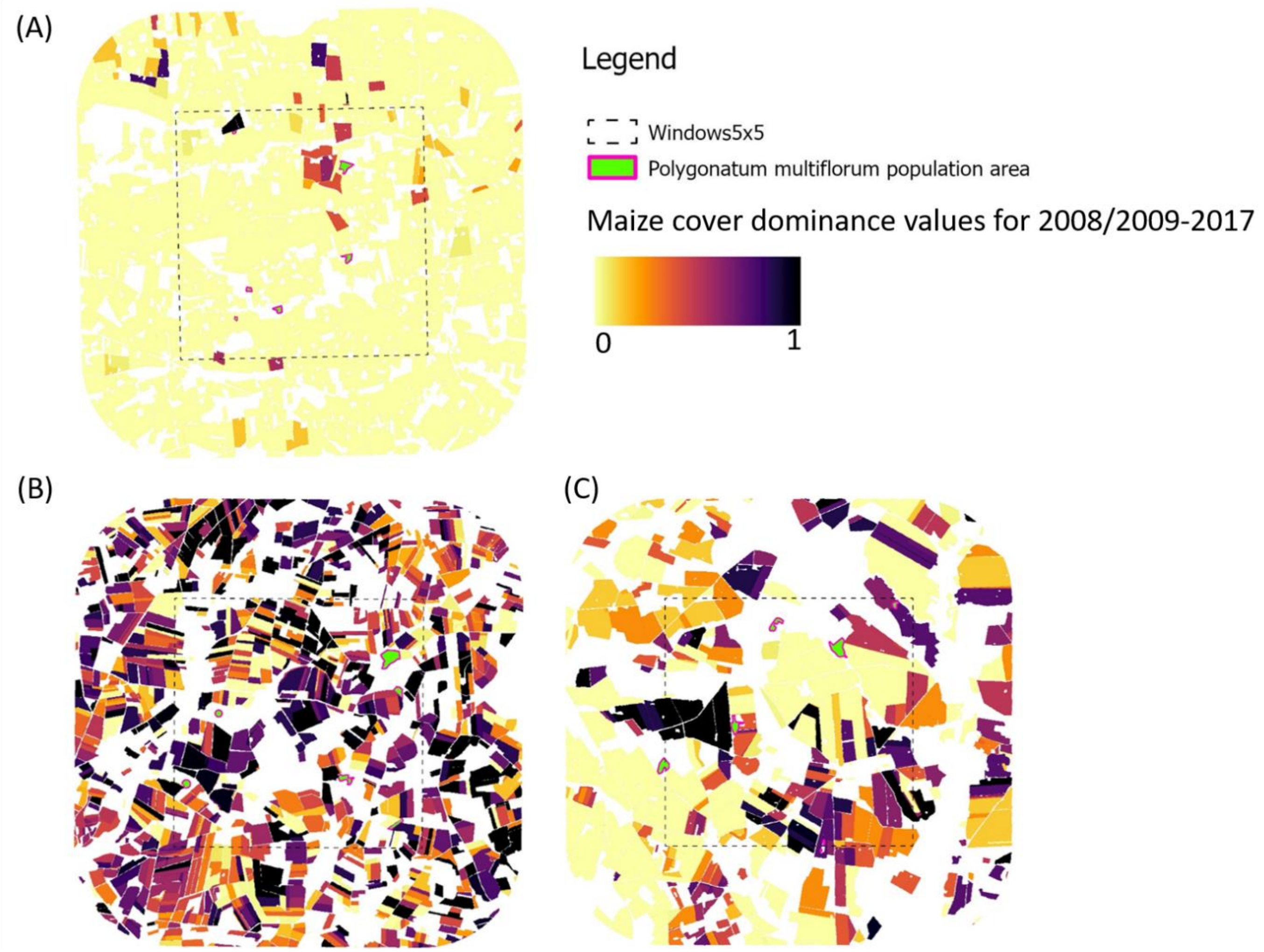

Figure S2.4: 7 km x 7 km maps around the 5 km x 5 km landscape windows in (A) Southern Sweden, (B) Eastern Germany, and (C) Western Germany. The maps show the degree of maize cover dominance per polygon.

S2.5 Rapeseed cover

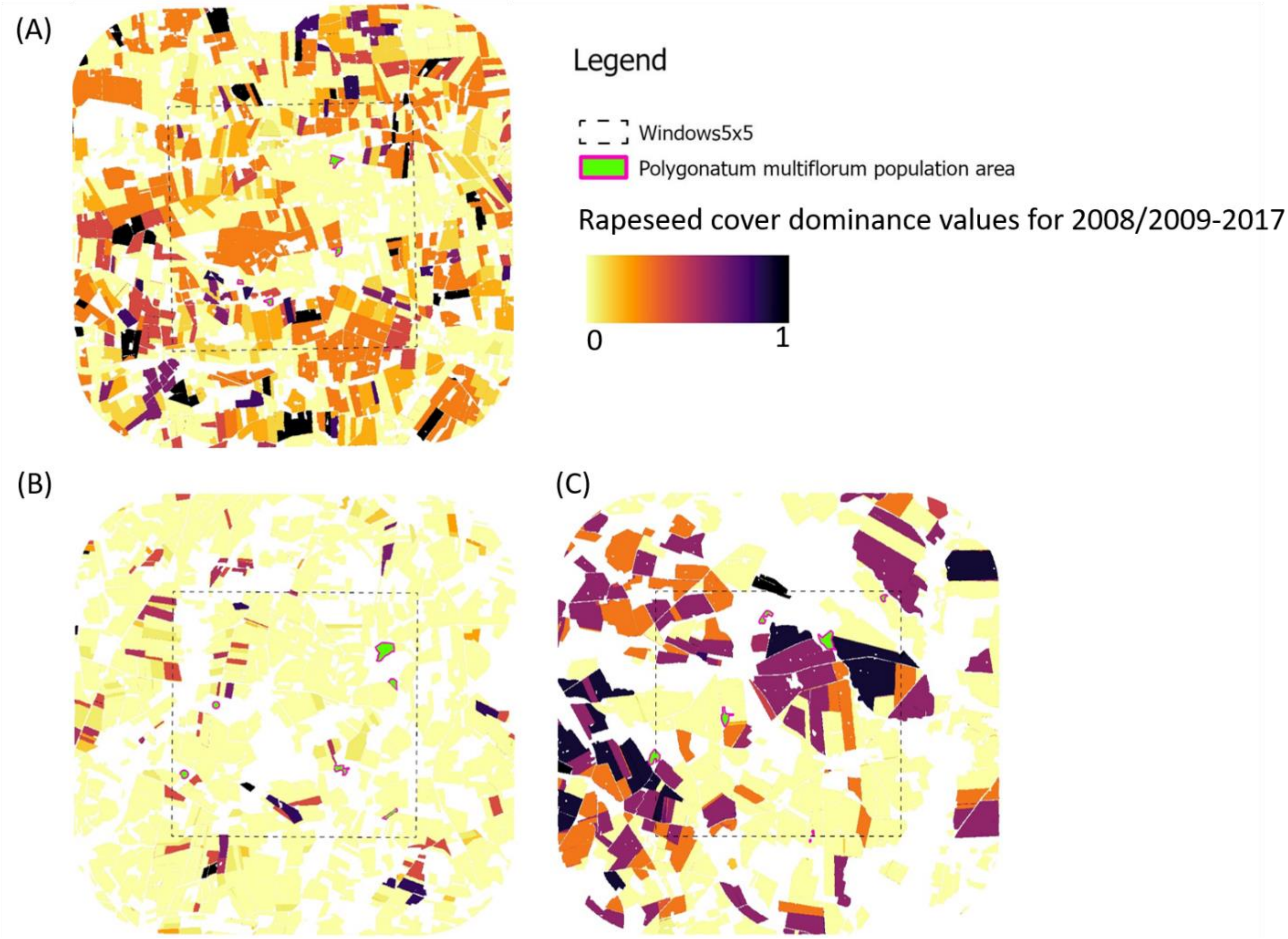

Figure S2.5: 7 km x 7 km maps around the 5 km x 5 km landscape windows in (A) Southern Sweden, (B) Eastern Germany, and (C) Western Germany. The maps show the degree of rapeseed cover dominance per polygon.

S2.6 Comparison of two periods of cover dominance

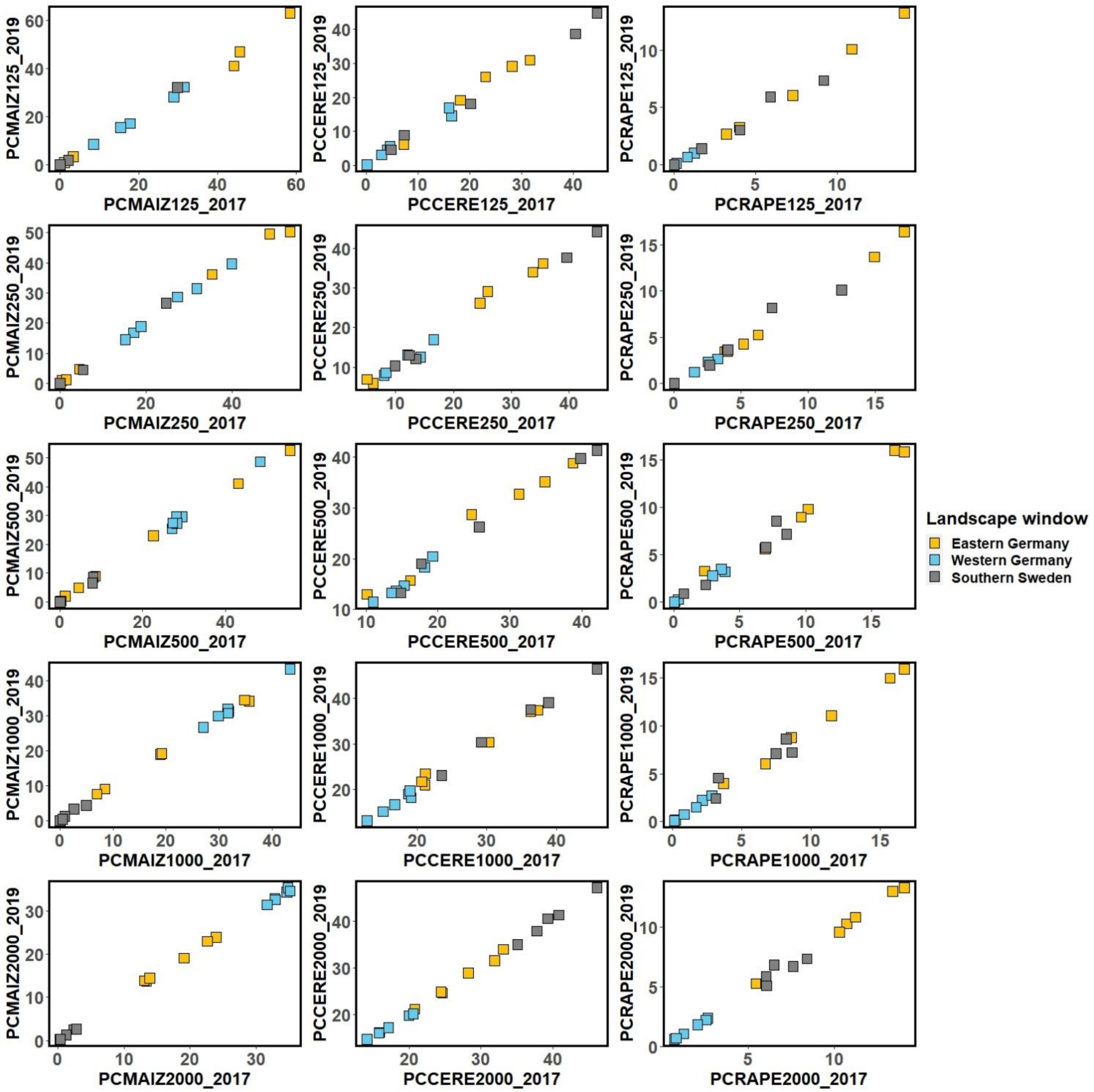

Figure S2.6: Correlation of two periods of crop dominance: Comparison of the crop dominance values of maize, cereals, and rapeseed between the period A: 2008/2009-2017 and the period B: 2008/2009-2019. Period A was used for modelling the forest herb's population genetic structure and period *b* for modelling the bumblebee's movement indicators.

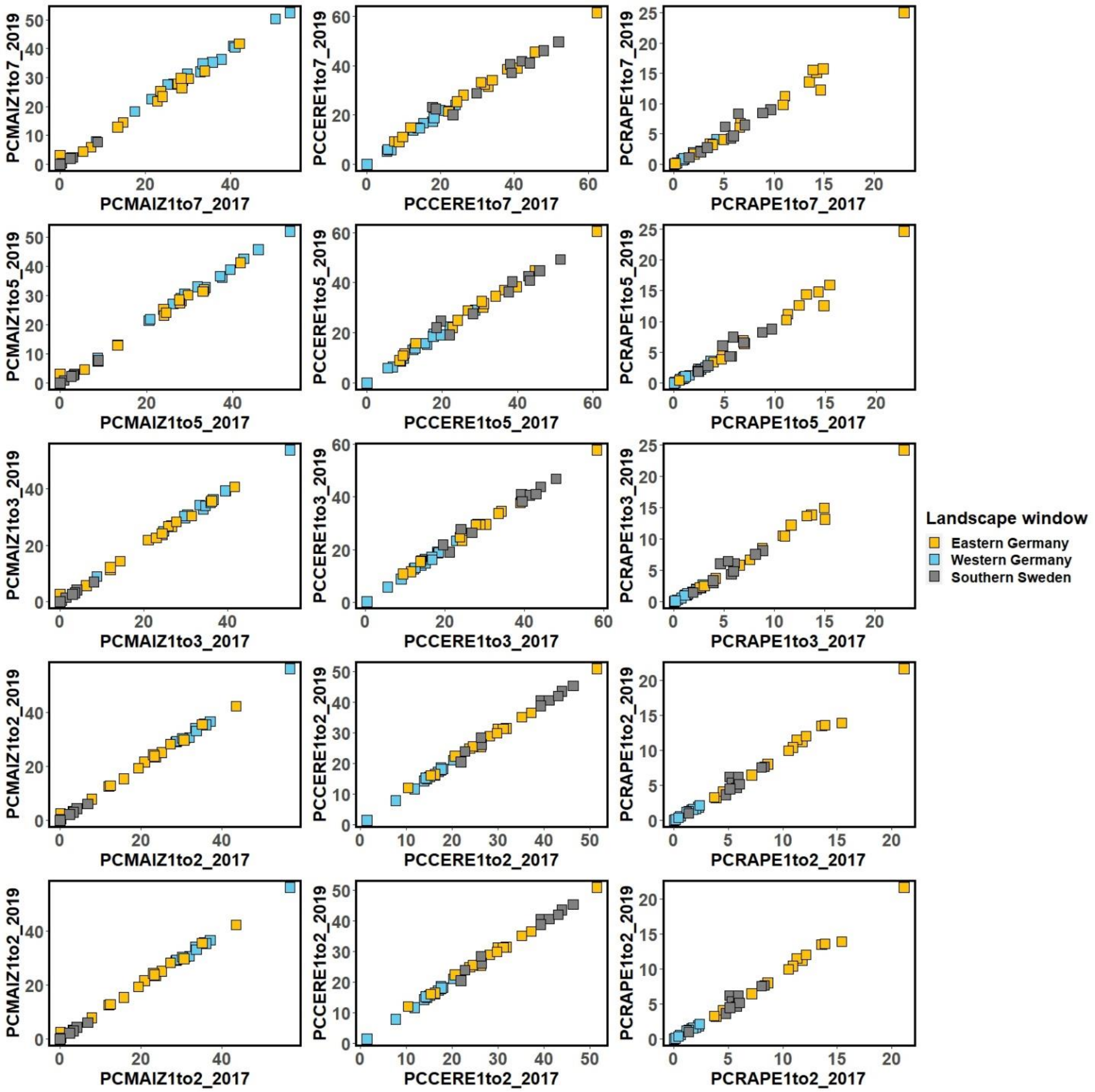

Figure S2.7: Correlation of two periods of crop dominance: Comparison of the crop dominance values of maize, cereals, and rapeseed between the period A: 2008/2009-2017 and the period B: 2008/2009-2019. Period A was used for modelling the forest herb's population genetic structure and period *b* for modelling the bumblebee's movement indicators.

S3 Bumblebee movement indicators

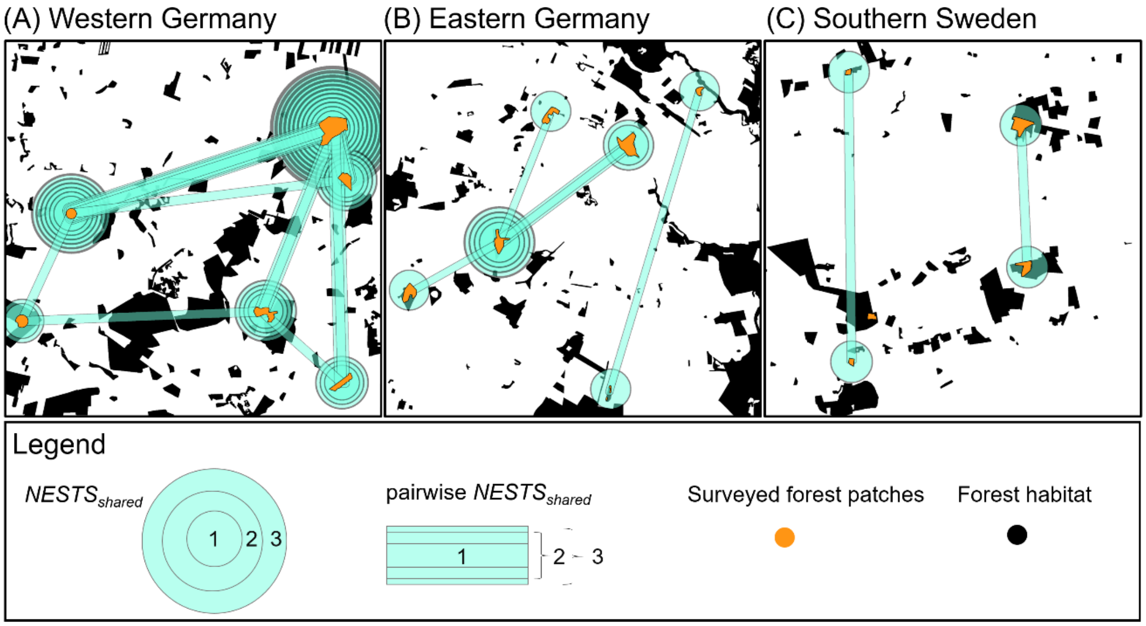

Figure S3.1: Distribution of shared nests (nests with individuals in different forest patches) of *B. pascuorum* within and among forest patches in three 5 km × 5 km landscape windows (A–C). The number of rectangles between two forest patches shows how many nests they shared. The number of circles show how many nests one forest patch shared with all other forest patches within the same landscape window.

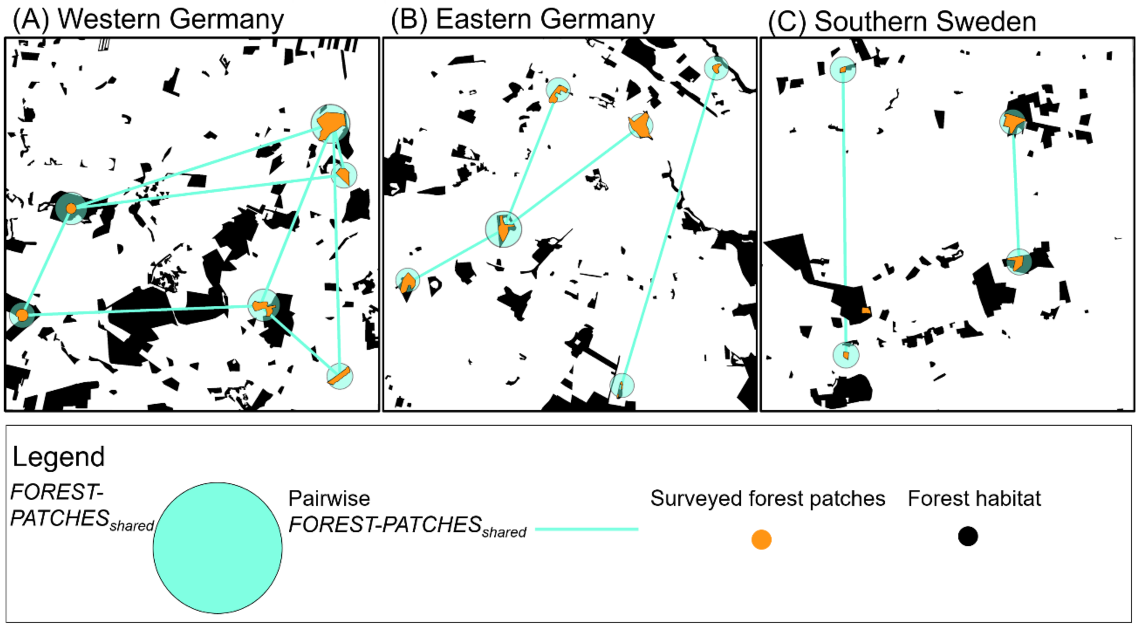

Figure S3.2: Distribution of forest patches that shared nests of *B. pascuorum* in the three 5 km x 5 km landscape windows (A-C).

S4 Measures of forest herb's genetic structure and linker's movement activity

Table S4.1: Overview of genetic diversity measures for *P. multiflorum* and bumblebee movement indicators for *B. pascuorum* at the node-level.

	Polygonatum multiflorum					***Bombus pascuorum***		
**Forest patch**	***n***	A_r	H_e	H_o	***F***	***n***	$NESTS_{shared}$	$\text{FOREST-PATCHES}_{SHARED}$
GeE01	20	6.56	0.72	0.78	-0.08	30	0.04	1
GeE04	20	10.28	0.77	0.83	-0.07	26	0.08	1
GeE07	20	6.86	0.65	0.67	-0.03	27	0.15	3
GeE10	20	7.41	0.67	0.73	-0.08	17	0.07	1
GeE48	20	9.64	0.74	0.8	-0.08	24	0.04	1
GeE51	20	8.07	0.74	0.83	-0.12	15	0.08	1
GeW01	19	11.17	0.74	0.76	-0.04	29	0.42	4
GeW02	10	6.67	0.74	0.8	-0.07	36	0.06	2
GeW03	20	8.89	0.77	0.79	-0.02	27	0.22	3
GeW09	20	9.34	0.75	0.73	0.02	25	0.13	2
GeW11	20	9.99	0.76	0.75	0.02	33	0.14	3
GeW15	14	8.83	0.75	0.8	-0.06	27	0.16	2
SwS05	20	7.87	0.74	0.87	-0.17	19	0.05	1
SwS13	19	7.83	0.75	0.81	-0.08	16	0.07	1
SwS21	20	8.2	0.74	0.87	-0.18	20	0.06	1
SwS38	20	6.54	0.70	0.88	-0.25	23	0	0
SwS44	20	8.36	0.74	0.83	-0.11	14	0.07	1

Table S4.2: Overview of genetic differentiation for *P. multiflorum* and bumblebee movement indicators for *B. pascuorum* at the link-level.

	Polygonatum multiflorum	***Bombus pascuorum***	
**Forest patch pairs**	***Proportion of shared alleles*** (D_{ps})		**Geographic distance [km]**
GeE01/GeE04	0.46	0.23	1.0
GeE01/GeE07	0.55	0.22	1.8
GeE01/GeE10	0.48	0.29	3.4
GeE01/GeE48	0.44	0.25	2.2
GeE01/GeE51	0.5	0.29	4.3
GeE04/GeE07	0.53	0.24	2.3
GeE04/GeE10	0.48	0.24	4.0
GeE04/GeE48	0.44	0.24	1.2
GeE04/GeE51	0.48	0.29	3.7
GeE07/GeE10	0.43	0.25	1.4
GeE07/GeE48	0.48	0.25	3.8
GeE07/GeE51	0.53	0.30	2.8

GeE10/GeE48	0.48	0.23	5.5
GeE10/GeE51	0.53	0.29	3.4
GeE48/GeE51	0.47	0.25	4.9
GeW01/GeW02	0.45	0.22	4.5
GeW01/GeW03	0.45	0.18	3.3
GeW01/GeW09	0.39	0.20	3.0
GeW01/GeW11	0.45	0.19	2.2
GeW01/GeW15	0.41	0.19	4.2
GeW02/GeW03	0.42	0.18	1.4
GeW02/GeW09	0.44	0.20	4.1
GeW02/GeW11	0.46	0.18	3.0
GeW02/GeW15	0.46	0.21	4.5
GeW03/GeW09	0.40	0.19	4.0
GeW03/GeW11	0.47	0.19	2.7
GeW03/GeW15	0.42	0.2	3.5
GeW09/GeW11	0.45	0.17	1.2
GeW09/GeW15	0.39	0.22	2.4
GeW11/GeW15	0.37	0.20	1.9
SwS05/SwS13	0.49	0.26	2.2
SwS05/SwS21	0.49	0.2	3.3
SwS05/SwS38	0.49	0.20	3.1
SwS05/SwS44	0.48	0.21	3.7
SwS13/SwS21	0.41	0.21	1.6
SwS13/SwS38	0.46	0.21	3.0
SwS13/SwS44	0.50	0.20	3.6
SwS21/SwS38	0.43	0.22	2.0
SwS21/SwS44	0.35	0.17	2.4
SwS38/SwS44	0.46	0.23	0.5

S5 Univariate models

Table S5: Overview of the univariate models of landscape metrics on the movement indicators of *B. pascuorum* with p < 0.15. At the node level, the optimum buffer size and at the link level the optimum length-to-width ratio is shown.

	NESTS$_{shared}$	***FOREST-PATCHES$_{shared}$***	***BomD$_{PS}$***
SEMNATGRASS	2000 (p=0.001)	2000 (p=0.0004)	1to3 (p=0.0446)
MAIZE	2000 (p=0.0016)	2000 (p=0.0002)	1to7 (p=0.0114)
CEREAL	1000 (p=0.0059)	2000 (p=0.0022)	
RAPESEED	2000 (p=0.0262)	2000 (p=0.0092)	
SEMNATVEG	2000 (p=0.0267)	2000 (p=0.1354)	
GRASS	2000 (p=0.0486)		2to3 (p=0.1269)
D_FOREST	500 (p=0.1108)	500 (p=0.1241)	
C_FOREST			
ORCHARD			
SETTLE			
L_FRINGE			2to3 (p=0.0679)
L_ROAD			
L_WOOD			
L_WATER	125 (p=0.0914)	125 (p=0.0149)	
EDGEDEN	125 (p=0.114)		2to3 (p=0.0839)
LANDHET	125 (p=0.0377)	125 (p=0.0446)	1to2 (p=0.031)

S6 Tests for collinearity

Table S6.1: Test for collinearity among all landscape metrics with a $p < 0.15$ in single-metric models for $NESTS_{shared}$. Red colors indicate a Pearson correlation of $|r| \geq 0.7$.

MAIZE2000	1	0.65	0.61	0.47	-0.34	-0.59	-0.93	-0.81	-0.2	-0.31
SEMNATVEG2000	0.65	1	0.19	0.12	0.11	-0.59	-0.49	-0.41	-0.25	-0.13
GRASS2000	0.61	0.19	1	0.37	-0.42	-0.34	-0.74	-0.72	0.27	-0.15
L_WATER125	0.47	0.12	0.37	1	-0.4	0.06	-0.47	-0.43	-0.01	-0.28
D_FOREST500	-0.34	0.11	-0.42	-0.4	1	-0.2	0.53	0.13	-0.27	-0.01
RAPESEED2000	-0.59	-0.59	-0.34	0.06	-0.2	1	0.55	0.76	-0.09	-0.13
SEMNATGRASS2000	-0.93	-0.49	-0.74	-0.47	0.53	0.55	1	0.8	-0.06	0.14
CEREALS1000	-0.81	-0.41	-074	-0.43	0.13	0.76	0.8	1	-0.06	0.15
EDGEDEN125	-0.2	-0.25	0.27	-0.01	-0.27	-0.09	-0.06	-0.06	1	0.41
LANDHET125	-0.31	-0.13	-0.15	-0.28	-0.01	-0.13	0.14	0.15	0.41	1
	MAIZE2000	SEMNATVEG2000	GRASS2000	L_WATER125	D_FOREST500	RAPESEED2000	SEMNATGRASS2000	CEREALS1000	EDGEDEN125	LANDHET125

Table S6.2: Test for collinearity among all landscape metrics with a $p < 0.15$ in single-metric models for *FOREST-PATCHES*$_{shared}$. Red colors indicate a Pearson correlation of $|r| \geq 0.7$.

	L_WATER125	MAIZE2000	SEMNATVEG2000	RAPESEED2000	SEMNATGRASS2000	CEREAL2000	LANDHET125	D_FOREST500
L_WATER125	1	0.47	0.12	0.06	-0.47	-0.38	-0.28	-0.4
MAIZE2000	0.47	1	0.65	-0.59	-0.93	-0.92	-0.31	-0.34
SEMNATVEG2000	0.12	0.65	1	-0.59	-0.49	-0.59	-0.13	0.11
RAPESEED2000	0.06	-0.59	-0.59	1	0.55	0.69	-0.13	-0.2
SEMNATGRASS2000	-0.47	-0.93	-0.49	0.55	1	0.91	0.14	0-53
CEREAL2000	-0.38	-0.92	-0.59	0.69	0.91	1	0.25	0.24
LANDHET125	-0.28	-0.31	-0.13	-0.13	0.14	0.25	1	-0.01
D_FOREST500	-0.4	-0.34	0.11	-0.2	0.53	0.24	-0.01	1

Table S6.3: Test for collinearity among all landscape metrics with a $p < 0.15$ in single-metric models for *BomD*$_{PS}$. Red colors indicate a Pearson correlation of $|r| \geq 0.7$.

	MAIZE1to7	LANDHET1to2	GRASS2to3	L_FRINGE2to3	SEMNATGRASS1to3	EDGEDEN2to3
MAIZE1to7	1	0.21	-0.07	-0.15	-0.66	-0.33
LANDHET1to2	0.21	1	-0.54	0.09	0.1	0.13
GRASS2to3	-0.07	-0.54	1	-0.36	-0.36	-0.11
L_FRINGE2to3	-.0.15	0.09	-0.36	1	0.4	0.45
SEMNATGRASS1to3	-0.66	0.1	-0.36	0.4	1	0.63
EDGEDEN2to3	-0.33	0.13	-0.11	0.45	0.63	1

S7 Comparison Step 2 Models and Step 3 Models

Table S7: Overview of the comparison between Step 2 Models and Step 3 Models. The table shows the count of Step 3 Models with a lower AICc than Step 2 Models, followed by the count of Step 3 Models with a higher *AICc* than Step 2 Models (denoted with ':'). The proportion of Step 3 Models 3 with a higher *AICc* than Step 2 Models is also shown in parentheses. The symbols indicate the used sets of landscape metrics:

■: *MAIZE2000 + LANDHET125,* □: *SEMINATGRASS2000 + EDGEDEN250 + EDGEDEN250²,* ●: *SEMINATGRASS1to3 + MAIZE1to7 + MAIZE1to7²,* ○: *SEMINATGRASS1to3 + MAIZE1to7*

A_r	■:	241 : 10,039 (< 97 %)	□:	2,837 : 7,443 (< 72 %)
H_e	■:	2,453 : 7,827 (< 76 %)	□:	4,605 : 5,675 (< 55 %)
H_o	■:	534 : 9,746 (< 94 %)	□:	11 : 10,269 (< 99 %)
F	■:	45 : 10,235 (< 99 %)	□:	304: 9,976 (< 97 %)
Pol D_{PS}	●:	8,219,415: 12,327,608 (59<%)	○:	6,325,714: 14,221,309 (69<%)

S8 Step 3 Models best

Table S8.1: Summary of 72 Step 3 Models best describing landscape effects on the population genetic structure (Pop.genstruct.) of *P. multiflorum*. At the node-level, allelic richness (A_r), expected (H_e) and observed heterozygosity (H_o), and the *F*-value are used as response variable and at the link-level $PolD_{PS}$. Presented are the included landscape metrics for each model, the marginal/conditional r^2 values, and the $\Delta AICc$ in comparison to the model with the lowest *AICc* among Step 3 Models best for the respective population genetic measure.

Step 3 model	Pop.gen struct.	Included landscape metrics<br>With regression coefficient and *p*-value			r^2	$\Delta AICc$
**Model 3 best a**	A_r	**LANDHET250**<br>*b* = -0.98, *p* = 0.0000	**D_FOREST125**<br>*b* = -0.62, *p* = 0.0003		0.55/0.87	0
**Model 3 best b**	A_r	**LANDHET250**<br>*b* = 1, *p* = 0.0000	**LANDHET250²**<br>*b* = -0.17, *p* = 0.0547	**D_FOREST125**<br>*b* = -0.63, *p* = 0.0001	0.58/0.90	0.04
**Model 3 best c**	A_r	**LANDHET500**<br>*b* = 0.64, *p* = 0.0017	**LANDHET500²**<br>*b* = -0.43, *p* = 0.0010	**GRASS2000**<br>*b* = 0.73, *p* = 0.0006	0.75/0.78	0.12
**Model 3 best d**	H_e	**L_WATER2000**<br>*b* = 0.55, *p* = 0.0043	**L_WATER2000²**<br>*b* = -0.61, *p* = 0.0089	**ORCHARD1000**<br>*b* = -0.56, *p* = 0.0057	0.65/0.65	0
**Model 3 best e**	H_e	**SEMANTVEG2000**<br>*b* = 0.97, *p* = 0.0009	**MAIZE1000**<br>*b* = -0.52, *p* = 0.0378		0.55/0.55	0.3
**Model 3 best f**	H_e	**SEMNATVEG2000**<br>*b* = 0.65 *p* = 0.0057			0.41/0.41	1.77
**Model 3 best g**	H_e	**SEMANTVEG2000**<br>*b* = 0.87, *p* = 0.0015	**MAIZE250**<br>*b* = -0.42, *p* = 0.0742		0.51/0.51	1.79
**Model 3 best h**	H_e	**SEMANTVEG2000**<br>*b* = 0.95, *p* = 0.0018	**MAIZE500**<br>*b* = -0.46, *p* = 0.0746		0.51/0.51	1.81
**Model 3 best i**	H_o	**MAIZE1000**<br>*b* = -0.85, *p* = 0.0003	**MAIZE1000²**<br>*b* = 0.43, *p* = 0.0474	**C_FOREST500**<br>*b* = 0.55, *p* = 0.0039	0.76/0.76	0
**Model 3 best j**	H_o	**MAIZE1000**<br>*b* = -0.51, *p* = 0.0048	**D_FOREST500**<br>*b* = 0.53, *p* = 0.0038		0.69/0.69	0.19
**Model 3 best k**	H_o	**MAIZE1000**<br>*b* = -0.99, *p* = 0.0001	**C_FOREST500**<br>*b* = 0.59, *p* = 0.0042		0.68/0.68	0.43
**Model 3 best l**	H_o	**MAIZE2000**<br>*b* = -1.02, *p* = 0.0002	**L_FRINGE250**<br>*b* = -0.62, *p* = 0.0043		0.67/0.7	0.69
**Model 3 best m**	H_o	**MAIZE2000**<br>*b* = -1.21, *p* = 0.0000	**C_FOREST250**<br>*b* = 1.34, *p* = 0.0014	**C_FOREST250²**<br>*b* = -0.76, *p* = 0.0066	0.74/0.74	1.23
**Model 3 best n**	***F***	**MAIZE2000**	**C_FOREST250**	**C_FOREST250²**	0.82/0.82	0

		b = 1.16, *p* = 0.0000	*b* = -1.14, *p* = 0.0010	*b* = 0.79, *p* = 0.0011		
**Model 3 best o**	***F***	**SEMANTGRASS2000**	**L_FRINGE500**		0.76/0.76	1.06
		b = -0.99, *p* = 0.0000	*b* = 0.43, *p* = 0.0083			
**Model 3 best p**	***F***	**SEMANTGRASS2000**	**C_FOREST125**	**C_FOREST125^2**	0.61/0.61	1.6
		b = -1.02, *p* = 0.0000	*b* = -0.92, *p* = 0.0053	*b* = 0.80, *p* = 0.0022		
**Model 3 best q**	***PolDps***	**SEMNATGRASS1to2**	**RAPESEED1to2**	**EDGEDEN**	0 .53/0 .66	0
		b = -0.55, *p* = 0.0041	*b* = -0.43, *p* = 0.0011	*b* = 0.02, *p* = 0.0030		
		**L_ROAD1to3**	**L_ROAD1to3^2**			
		b = -0.01, *p* = 0.0057	*b* = -0.01, *p* = 0.0001			
**Model 3 best r**	***PolDps***	**ORCHARD2to3**	**MAIZE2to3**	**L_WOOD2to3**	0 .63/0 .63	0.02
		b = -0.43, *p* = 0.0021	*b* = 0.00, *p* = 0.0000	*b* = 0.01, *p* = 0.0006		
		**L_ROAD1to2**	**L_ROAD1to2^2**			
		b = -0.01, *p* = 0.0057	*b* = -0.01, *p* = 0.0001			
**Model 3 best s**	***PolDps***	**RAPESEED2to3**	**L_WATER2to3**	**EDGEDEN1to2**	0 .51/0 .64	0.15
		b = 0.00, *p* = 0.0266	*b* = -0.01, *p* = 0.0139	*b* = 0.2, *p* = 0.0068		
		**L_ROAD1to2**	**L_ROAD1to2^2**			
		b = -0.02, *p* = 0.0002	*b* = -0.01, *p* = 0.0000			
**Model 3 best t**	***PolDps***	**RAPESEED2to3**	**L_WATER2to3**	**EDGEDEN2to3**	0 .43/0 .68	0.18
		b = 0.01, *p* = 0.0496	*b* = -0.01, *p* = 0.0116	*b* = 0.2, *p* = 0.0037		
		**L_ROAD1to2**	**L_ROAD1to2^2**			
		b = -0.02, *p* = 0.0001	*b* = -0.01, *p* = 0.0000			
**Model 3 best u**	***PolDps***	**CEREAL2to3**	**L_ROAD1to2**	**ORCHARD1to3**	0 .65/0 .65	0.40
		b = 0.00, *p* = 0.0001	*b* = -0.02, *p* = 0.0000	*b* = -0.00, *p* = 0.3135		
		**ORCHARD1to3^2**	**L_WOOD1to5**	**L_WOOD1to5^2**		
		b = 0.01, *p* = 0.0056	*b* = -0.00, *p* = 0.8031	*b* = -0.01, *p* = 0.0049		
**Model 3 best v**	***PolDps***	**CEREAL1to2**	**L_ROAD1to2**	**ORCHARD1to3**	0 .65/0 .65	0.61
		b = 0.01, *p* = 0.0002	*b* = -0.02, *p* = 0.0000	***b*** = -0.00, *p* = 0.2966		
		**ORCHARD1to3^2** ***b*** =	**L_WOOD1to5**	**L_WOOD1to5^2**		
		0.01, *p* = 0.0053	*b* = 0.00, *p* = 0.8624	*b* = -0.01, *p* = 0.0070		
**Model 3 best w**	***PolDps***	**ORCHARD2to3**	**MAIZE2to3**	**L_ROAD1to2**	0 .54/0 .59	0.77
		b = 0.01, *p* = 0.0049	*b* = -0.00, *p* = 0.0036	*b* = -0.01, *p* = 0.0027		
		**L_ROAD1to2^2**				
		b = -0.01, *p* = 0.0003				
**Model 3 best x**	***PolDps***	**RAPESEED2to3**	**L_WATER1to2**	**EDGEDEN1to2**	0 .42/0 .69	0.78
		b = 0.01, *p* = 0.0480	b = -0.01, *p* = 0.0098	*b* = 0.02, *p* = 0.0034		
		**L_ROAD1to2**	**L_ROAD1to2^2**			
		b = -0.03, *p* = 0.0001	*b* = -0.01, *p* = 0.0000			
**Model 3 best y**	***PolDps***	**ORCHARD2to3**	**MAIZE2to3**	**L_ROAD1to2**	0 .64/0 .64	0.86
		b = 0.01, *p* = 0.0004	*b* = -0.00, *p* = 0.0001	*b* = -0.01, *p* = 0.0027		

		**L_ROAD1to2^2**	**L_WATER1to5**	**L_WATER1to5^2**		
		b = -0.01, *p* = 0.0754	*b* = 0.00, *p* =0.7977	*b* = -0.01, *p* = 0.0211		
**Model 3 best z**	***PolDps***	**CEREAL2to3**	**L_ROAD1to2**	**GRASS1to5**	0 .64/0 .64	0.87
		b = 0.00, *p* = 0.0038	*b* = -0.02, *p* = 0.0000	*b* = -0.01, *p* = 0.1077		
		**GRASS1to5^2**	**ORCHARD1to2**	**ORCHARD1to2^2**		
		b = -0.01, *p* = 0.0077	*b* = 0.00, *p* = 0.4467	*b* = 0.01, *p* = 0.0080		
**Model 3 best aa**	***PolDps***	**MAIZE2to3**	**L_ROAD1to2**	**GRASS1to5**	0 .64/0 .64	1.06
		b = -0.00, *p* = 0.0041	*b* = -0.02, *p* = 0.0000	*b* = -0.01, *p* = 0.0002		
		**GRASS1to5^2**	**ORCHARD1to2**	**ORCHARD1to2^2**		
		b = -0.01, *p* = 0.0012	*b* = 0.00 *p* = 0.3195	*b* = 0.01, *p* = 0.0013		
**Model 3 best ab**	***PolDps***	**CEREAL2to3**	**L_ROAD1to2**	**LANDHET2to3**	0 .62/0 .62	1.07
		b = 0.00, *p* = 0.0000	*b* = -0.02, *p* = 0.0000	*b* = 0.01, *p* = 0.0211		
		**ORCHARD1to2**	**ORCHARD1to2^2**			
		b = -0.00, p = 0.8171	*b* = 0.01, *p* = 0.0156			
**Model 3 best ac**	***PolDps***	**RAPESEED1to2**	**L_WATER2to3**	**EDGEDEN1to2**	0 .45/0 .66	1.08
		b = 0.01, *p* = 0.0495	*b* = -0.02, *p* = 0.0088	*b* = 0.02, *p* = 0.0044		
		**L_ROAD1to2**	**L_ROAD1to2^2**			
		b = -0.02, *p* = 0.0001	*b* = -0.01, *p* = 0.0000			
**Model 3 best ad**	***PolDps***	**MAIZE1to3**<br>*b* = -0.01, *p* =0.0103	**L_ROAD1to2**<br>*b* = -0.02, *p* = 0.0000	**GRASS1to5**<br>*b* = -0.01, *p* = 0.0001	0 .62/0 .64	1.10
		**GRASS1to5^2**	**ORCHARD1to2**	**ORCHARD1to2^2**		
		b = -0.01, *p* = 0.0010	*b* = 0.00, *p* = 0.3213	b = 0.01, p = 0.0022		
**Model 3 best ae**	***PolDps***	**ORCHARD2to3**<br>*b* = 0.01, *p* = 0.0192	**MAIZE2to3**<br>*b* = -0.00, *p* = 0.0020	**L_WATER2to3**<br>*b* = -0.01, *p* =0.1122	0 .62/0 .62	1.11
		**L_ROAD1to2**<br>*b* = -0.01, *p* = 0.0003	**L_ROAD1to2^2**<br>*b* = -0.01, *p* = 0.0001			
**Model 3 best af**	***PolDps***	**D_FOREST2to3**<br>*b* = -0.02, *p* =0.0001	**LANDHET2to3**<br>*b* = 0.01, *p* = 0.0122	**SEMNATVEG2to3**<br>*b* = -0.01, *p* = 0.0007	0 .59/0 .64	1.14
		**SEMNATVEG2to3^2**<br>*b* = 0.01, *p* = 0.0154	**L_ROAD1to2**<br>*b* = -0.01, *p* = 0.0470	**L_ROAD1to2^2**<br>*b* = -0.01, *p* = 0.0009		
**Model 3 best ag**	***PolDps***	**CEREAL1to2**<br>*b* = 0.01, *p* = 0.0001	**L_ROAD1to2**<br>*b* = -0.02, *p* = 0.0000	**ORCHARD1to3**<br>*b* = -0.01, *p* =0.2713	0 .64/0 .64	1.15
		**ORCHARD1to3^2**<br>*b* = -0.01, *p* = 0.0049	**L_WOOD1to3**<br>*b* = 0.00, *p* = 0.6537	**L_WOOD1to3^2**<br>*b* = -0.01, *p* = 0.0083		
**Model 3 best ah**	***PolDps***	**ORCHARD2to3**<br>*b* = 0.02, *p* = 0.0000	**MAIZE2to3**<br>*b* = -0.00, *p* = 0.0003	**L_ROAD1to2**<br>*b* = -0.01, *p* = 0.0055	0 .62/0 .62	1.18
		**L_WATER1to5**<br>*b* = 0.00, *p* =0.5504	**L_WATER1to5^2**<br>*b* = -0.01, *p* = 0.0001			

**Model 3 best ai**	***PolDps***	**ORCHARD2to3**<br>*b* = 0.01, *p* = 0.0058	**MAIZE1to2**<br>*b* = -0.01, *p* = 0.0039	**L_WOOD2to3**<br>*b* = 0.01p = 0.0440	0 .62/0 .62	1.21
		**L_ROAD1to2**<br>*b* = -0.01, *p* = 0.0013	**L_ROAD1to2**^{2}<br>*b* = -0.01, *p* = 0.0002			
**Model 3 best aj**	***PolDps***	**MAIZE1to2**<br>*b* = -0.01, *p* =0.0076	**L_ROAD1to2**<br>*b* = -0.02, *p* = 0.0000	**GRASS1to5**<br>*b* = -0.01, *p* = 0.0001	0 .63/0 .64	1.21
		**GRASS1to5**^{2}<br>*b* = -0.01, *p* =0.0010	**ORCHARD1to2**<br>*b* = -0.00, *p* = 0.3287	**ORCHARD1to2**^{2}<br>*b* = 0.01, *p* = 0.0014		
**Model 3 best al**	***PolDps***	**ORCHARD2to3**<br>*b* = 0.01, *p* = 0.0069	**MAIZE2to3**<br>*b* = -0.00, *p* = 0.0027	**L_WOOD1to2**<br>*b* = 0.01, *p* = 0.1255	0 .61/0 .61	1.31
		**L_ROAD1to2**<br>*b* = -0.01, *p* =0.0010	**L_ROAD1to2**^{2}<br>*b* = -0.01, *p* = 0.0002			
**Model 3 best am**	***PolDps***	**RAPESEED2to3**<br>*b* = 0.01, *p* = 0.0033	**L_WATER2to3**<br>*b* = -0.01, *p* = 0.0241	**EDGEDEN1to3**<br>*b* = 0.01, *p* = 0.0149	0 .61/0 .61	1.32
		**L_ROAD1to2**<br>*b* = -0.02, *p* =0.0004	**L_ROAD1to2**^{2}<br>*b* = -0.01, *p* = 0.0001			
**Model 3 best an**	***PolDps***	**RAPESEED2to3**<br>*b* = 0.01, *p* = 0.0017	**L_WATER1to2**<br>*b* = -0.01, *p* = 0.0242	**EDGEDEN1to3**<br>*b* = 0.01, *p* = 0.0111	0 .61/0 .61	1.33
		**L_ROAD1to2**<br>*b* = -0.02, *p* =0.0005	**L_ROAD1to2**^{2}<br>*b* = -0.01, *p* = 0.0001			
**Model 3 best ao**	***PolDps***	**D_FOREST2to3**<br>*b* = -0.02, *p* =0.0000	**SEMNATVEG2to3**<br>*b* = -0.02, *p* = 0.0003	**SEMNATVEG2to3**^{2}<br>*b* = 0.01, *p* = 0.0093	0 .62/0 .67	1.37
		**L_ROAD1to2**<br>*b* = -0.01, *p* = 0.0129	**L_ROAD1to2**^{2}<br>*b* = -0.01, *p* = 0.0011	**LANDHET2to3**<br>*b* = 0.01, *p* = 0.0043		
		**LANDHET2to3**^{2}<br>*b* = -0.00, *p* = 0.0842				
**Model 3 best ap**	***PolDps***	**CEREAL2to3**<br>*b* = 0.00, *p* = 0.0000	**L_ROAD1to2**<br>*b* = -0.02, *p* = 0.0000	**LANDHET2to3**<br>*b* = 0.01, *p* = 0.0251	0 .61/0 .61	1.38
		**ORCHARD1to3**<br>*b* = -0.00, *p* = 0.3684	**ORCHARD1to3**^{2}<br>*b* = 0.01, *p* = 0.0143			
**Model 3 best aq**	***PolDps***	**MAIZE2to3**<br>*b* = -0.00, *p* = 0.0009	**GRASS2to3**<br>*b* = -0.01, *p* = 0.0132	**GRASS2to3**^{2}<br>*b* = -0.01, *p* = 0.0223	0 .66/0 .66	1.39
		**ORCHARD1to2**<br>*b* = 0.00, *p* =0.7061	**ORCHARD1to2**^{2}<br>*b* = 0.01, *p* = 0.0084	**L_ROAD1to2**<br>*b* = -0.02, *p* = 0.0000		

		**L_ROAD1to2^2**<br>b = -0.01, p =0.0362				
**Model 3 best ar**	***PolDps***	**GRASS1to2**<br>b = -0.02, p =0.0000	**SEMNATVEG1to3**<br>b = -0.01, p = 0.0663	**SEMNATVEG1to3^2**<br>b = 0.01, p = 0.0096	0 .36/0 .67	1.42
		**MAIZE2to3**<br>b = 0.00, p = 0.1441	**MAIZE2to3^2**<br>b = -0.00, p = 0.0150	**L_WATER1to5**<br>b = 0.01, p = 0.0111		
		**L_WATER1to5^2**<br>b = -0.01, p =0.0022				
**Model 3 best as**	***PolDps***	**CEREAL1to2**<br>b = 0.01, p = 0.0049	**L_ROAD1to2**<br>b = -0.02, p = 0.0000	**GRASS1to5**<br>b = -0.01, p = 0.1979	0 .64/0 .64	1.49
		**GRASS1to5^2**<br>b = -0.01, p =0.0106	**ORCHARD1to2**<br>b = 0.00, p = 0.4742	**ORCHARD1to2^2**<br>b = 0.01, p = 0.0083		
**Model 3 best at**	***PolDps***	**RAPESEED2to3**<br>b = 0.01, p = 0.0030	**L_WATER1to3**<br>b = -0.01, p = 0.0261	**EDGEDEN1to3**<br>b = 0.01, p = 0.0100	0 .61/0 .61	1.49
		**L_ROAD1to2**<br>b = -0.02, p =0.0006	**L_ROAD1to2^2**<br>b = -0.01, p = 0.0001			
**Model 3 best au**	***PolDps***	**RAPESEED2to3**<br>b = 0.01, p = 0.0345	**L_WATER1to2**<br>b = -0.01, p = 0.0249	**EDGEDEN2to3**<br>b = 0.02, p = 0.0060	0 .42/0 .67	1.50
		**L_ROAD1to2**<br>b = -0.02, p =0.0002	**L_ROAD1to2^2**<br>b = -0.01, p = 0.0000			
**Model 3 best av**	***PolDps***	**RAPESEED1to3**<br>b = 0.01, p = 0.0036	**L_WATER2to3**<br>b = -0.01, p = 0.0096	**EDGEDEN1to3**<br>b = 0.01, p = 0.0121	0 .61/0 .61	1.52
		**L_ROAD1to2**<br>b = -0.02, p =0.0002	**L_ROAD1to2^2**<br>b = -0.01, p = 0.0002			
**Model 3 best aw**	***PolDps***	**CEREAL1to2**<br>b = 0.02, p = 0.0000	**L_ROAD1to2**<br>b = -0.01, p = 0.0001	**LANDHET2to3**<br>b = 0.01, p = 0.0398	0 .61/0 .61	1.62
		**ORCHARD1to2**<br>b = -0.00, p =0.8463	**ORCHARD1to2^2,**<br>p = 0.01, p = 0.0148			
**Model 3 best ax**	***PolDps***	**MAIZE2to3**<br>b = -0.00, p =0.0027	**GRASS1to5**<br>b = -0.01, p = 0.0176	**GRASS1to5^2**<br>b = -0.01, p = 0.0139	0 .65/0 .66	1.62
		**ORCHARD1to2**<br>b = 0.00, p = 0.4284	**ORCHARD1to2^2**<br>b = 0.01, p = 0.0044	**L_ROAD1to2**<br>b = -0.02, p = 0.0001		
		**L_ROAD1to2^2**<br>b = -0.00, p =0.1026				
**Model 3 best ay**	***PolDps***	**RAPESEED1to2**<br>b = 0.01, p = 0.0854	**L_WATER2to3**<br>b = -0.02, p = 0.0085	**EDGEDEN2to3**<br>b = 0.02, p = 0.0030	0 .38/0 .7	1.62

		**L_ROAD1to2**<br>*b* = -0.02, *p* =0.0001	**L_ROAD1to2^2**<br>*b* = -0.01, *p* = 0.0000			
**Model 3 best az**	***PolDps***	**RAPESEED2to3**<br>*b* = 0.01, *p* = 0.0000	**EDGEDEN2to3**<br>*b* = 0.02, *p* = 0.0054	**SEMNATGRASS1to2**<br>*b* = -0.02, *p* = 0.0106	0 .57/0 .64	1.64
		**SEMNATGRASS1to2^2**<br>*b* = 0.00, *p* = 0.4537	**L_ROAD1to3**<br>*b* = -0.01, *p* = 0.0078	**L_ROAD1to3^2^2**<br>*b* = -0.01, *p* = 0.0001		
**Model 3 best ba**	***PolDps***	**ORCHARD2to3**<br>*b* = 0.01, *p* = 0.0048	**CEREAL1to2**<br>*b* = 0.01, *p* = 0.0001	**L_ROAD1to2**<br>*b* = -0.01, *p* = 0.0003	0 .56/0 .56	1.65
**Model 3 best bb**	***PolDps***	**RAPESEED1to3**<br>*b* = 0.01, *p* = 0.0502	**L_WATER2to3**<br>*b* = -0.02, *p* = 0.0058	**EDGEDEN1to2**<br>*b* = 0.02, *p* = 0.0031	0 .42/0 .68	1.65
		**L_ROAD1to2**<br>*b* = -0.02, *p* = 0.0001	**L_ROAD1to2^2**<br>*b* = -0.01, *p* = 0.0000			
**Model 3 best bc**	***PolDps***	**CEREAL1to2**<br>*b* = 0.02, *p* = 0.0000	**L_ROAD1to2**<br>*b* = -0.01, *p* = 0.0000	**LANDHET2to3**<br>*b* = 0.01, *p* = 0.0513	0 .61/0 .61	1.67
		**ORCHARD1to3**<br>*b* = -0.00, *p* = 0.3715	**ORCHARD1to3^2**<br>*b* = 0.01, *p* = 0.0123			
**Model 3 best bd**	***PolDps***	**RAPESEED1to2**<br>*b* = 0.01, *p* = 0.0040	**L_WATER2to3**<br>*b* = -0.01, *p* = 0.0176	**EDGEDEN1to3**<br>*b* = 0.01, *p* = 0.0136	0 .61/0 .61	1.71
		**L_ROAD1to2**<br>*b* = -0.02, *p* = 0.0003	**L_ROAD1to2^2**<br>*b* = -0.01, *p* = 0.0002			
**Model 3 best be**	***PolDps***	**CEREAL2to3**<br>*b* = 0.00, *p* = 0.0002	**L_ROAD1to2**<br>*b* = -0.02, *p* = 0.0000	**ORCHARD1to3**<br>*b* = -0.00, *p* = 0.3457	0 .64/0 .64	1.74
		**ORCHARD1to3^2**<br>*b* = 0.01, *p* = 0.0072	**L_WOOD1to7**<br>*b* = -0.00, *p* = 0.8821	**L_WOOD1to7^2**<br>*b* = 0.01, *p* = 0.0105		
**Model 3 best bf**	***PolDps***	**D_FOREST1to2**<br>*b* = -0.00, *p* =0.1864	**ORCHARD2to3**<br>*b* = 0.01, *p* = 0.0041	**MAIZE2to3**<br>*b* = -0.00, *p* = 0.0077	0 .58/0 .6	1.74
		**L_ROAD1to2**<br>*b* = -0.01, *p* = 0.0020	**L_ROAD1to2^2**<br>*b* = -0.01, *p* = 0.0057			
**Model 3 best bg**	***PolDps***	**D_FOREST1to2**<br>*b* = -0.02, *p* = 0.0001	**LANDHET2to3**<br>*b* = 0.01, *p* = 0.0339	**SEMNATVEG2to3**<br>*b* = -0.01, *p* = 0.0020	0 .59/0 .66	1.76
		**SEMNATVEG2to3^2**<br>*b* = 0.01, *p* = 0.0171	**L_ROAD1to2**<br>*b* = -0.01, *p* = 0.0315	**L_ROAD1to2^2**<br>*b* = -0.01, *p* = 0.0008		
**Model 3 best bh**	***PolDps***	**ORCHARD2to3**	**CEREAL1to2**	**L_ROAD1to2**	0 .59/0 .59	1.80

		b = 0.01, *p* = 0.0065	*b* = 0.02, *p* = 0.0000	*b* = -0.01, *p* = 0.0001		
		**LANDHET1to7**<br>*b* = 0.01, *p* = 0.1067				
**Model 3 best bi**	***PolDps***	**CEREAL2to3**<br>*b* = 0.00, *p* = 0.0000	**L_ROAD1to2**<br>*b* = -0.01, *p* = 0.0001	**LANDHET2to3**<br>*b* = 0.01, *p* = 0.0413	0 .61/0 .61	1.86
		**ORCHARD2to3**<br>*b* = 0.00, *p* = 0.3326	**ORCHARD2to3^2**<br>*b* = 0.01, *p* = 0.0844			
**Model 3 best bj**	***PolDps***	**RAPESEED2to3**<br>*b* = 0.01, *p* = 0.0520	**L_WATER1to3**<br>*b* = -0.01, *p* = 0.0259	**EDGEDEN1to2**<br>*b* = 0.02, *p* = 0.0084	0 .44/0 .64	1.89
		**L_ROAD1to2**<br>*b* = -0.02, *p* = 0.0004	**L_ROAD1to2^2**<br>*b* = -0.01, *p* = 0.0001			
**Model 3 best bl**	***PolDps***	**CEREAL1to2**<br>*b* = 0.01, *p* = 0.0032	**L_ROAD1to2**<br>*b* = -0.02, *p* = 0.0000	**GRASS2to3**<br>*b* = -0.01, *p* = 0.0791	0 .63/0 .63	1.89
		**GRASS2to3^2**<br>*b* = -0.01, *p* = 0.0450	**ORCHARD1to3**<br>*b* = -0.00, *p* = 0.4030	**ORCHARD1to3^2**<br>*b* = 0.01, *p* = 0.0067		
**Model 3 best bm**	***PolDps***	**D_FOREST2to3**<br>*b* = -0.00, *p* =0.2007	**ORCHARD2to3**<br>*b* = 0.01, *p* = 0.0033	**MAIZE2to3**<br>*b* = -0.00, *p* = 0.0081	0 .57/0 .6	1.93
		**L_ROAD1to2**<br>*b* = -0.01, *p* = 0.0026	**L_ROAD1to2^2**<br>*b* = -0.01, *p* = 0.0059			
**Model 3 best bn**	***PolDps***	**ORCHARD2to3**<br>*b* = 0.01, *p* = 0.0037	**CEREAL2to3**<br>*b* = 0.00, *p* = 0.0001	**L_ROAD1to2**<br>*b* = -0.01, *p* = 0.0002	0 .56/0 .56	1.93
**Model 3 best bo**	***PolDps***	**ORCHARD2to3**<br>*b* = 0.01, *p* = 0.0105	**MAIZE1to3**<br>*b* = -0.01, *p* = 0.0097	**L_WOOD2to3**<br>*b* = 0.01, *p* = 0.0554	0 .59/0 .6	1.94
		**L_ROAD1to2**<br>*b* = -0.01, *p* = 0.0030	**L_ROAD1to2^2**<br>*b* = -0.01, *p* = 0.0002			
**Model 3 best bp**	***PolDps***	**ORCHARD2to3**<br>*b* = 0.01, *p* = 0.0051	**CEREAL2to3**<br>*b* = 0.00, *p* = 0.0000	**L_ROAD1to2**<br>*b* = -0.01, *p* 0.0001	0 .58/0 .58	1.95
		**LANDHET1to7**<br>*b* = 0.01 *p* = 0.1006				
**Model 3 best bq**	***PolDps***	**L_WOOD1to2**<br>*b* = 0.01, *p* = 0.0223	**L_ROAD1to2**<br>*b* = -0.02, *p* = 0.0000	**GRASS1to5**<br>*b* = -0.01, *p* = 0.0004	0 .62/0 .64	1.95
		**GRASS1to5^2**<br>*b* = -0.01, *p* = 0.0029	**ORCHARD1to2**<br>*b* = 0.00, *p* = 0.4689	**ORCHARD1to2^2)**<br>*b* = 0.01, *p* = 0.0040		
**Model 3 best br**	***PolDps***	**RAPESEED1to3**	**L_WATER1to2**	**EDGEDEN1to3**	0 .56/0 .6	1.96

		b = 0.01, *p* = 0.0105	*b* = -0.014, *p* = 0.0137	*b* = 0.02, *p* = 0.0090		
		L_ROAD1to2<br>*b* = -0.02, *p* = 0.0003	**L_ROAD1to2^2**<br>*b* = -0.01, *p* = 0.0001			
**Model 3 best bs**	***PolDps***	**ORCHARD2to3**<br>*b* = 0.01, *p* = 0.0525	**MAIZE2to3**<br>*b* = -0.00, *p* = 0.0120	**RAPESEED2to3**<br>*b* = 0.00, *p* = 0.1803	0 .61/0 .61	1.98
		**L_ROAD1to2**<br>*b* = -0.01, *p* = 0.0021	**L_ROAD1to2^2**<br>*b* = -0.01, *p* = 0.0008			
**Model 3 best bt**	***PolDps***	**CEREAL2to3**<br>*b* = 0.00p = 0.0002	**L_ROAD1to2**<br>*b* = -0.02, *p* = 0.0000	**ORCHARD1to3**<br>*b* = -0.00, *p* = 0.3009	0 .63/0 .63	1.99
		**ORCHARD1to3^2**<br>*b* = 0.01, *p* = 0.0054	**L_WOOD1to3**<br>*b* = 0.00, *p* = 0.9529	**L_WOOD1to3^2**<br>*b* = -0.01, *p* = 0.0072		
**Model 3 best bu**	***PolDps***	**RAPESEED1to2**<br>*b* = 0.01, *p* = 0.0022	**L_WATER1to2**<br>*b* = -0.01, *p* = 0.0201	**EDGEDEN1to3**<br>*b* = 0.01, *p* = 0.0107	0 .61/0 .61	1.99
		**L_ROAD1to2**<br>*b* = -0.02, *p* = 0.0004	**L_ROAD1to2^2**<br>*b* = -0.01, *p* = 0.0002			
**Model 3 best bv**	***PolDps***	**ORCHARD2to3**<br>*b* = 0.01, *p* = 0.0340	**CEREAL2to3**<br>*b* = 0.00, *p* = 0.0000	**L_ROAD1to2**<br>*b* = -0.02, *p* = 0.0001	0 .58/0 .58	1.99
		**LANDHET2to3**<br>*b* = 0.01 0.1045				

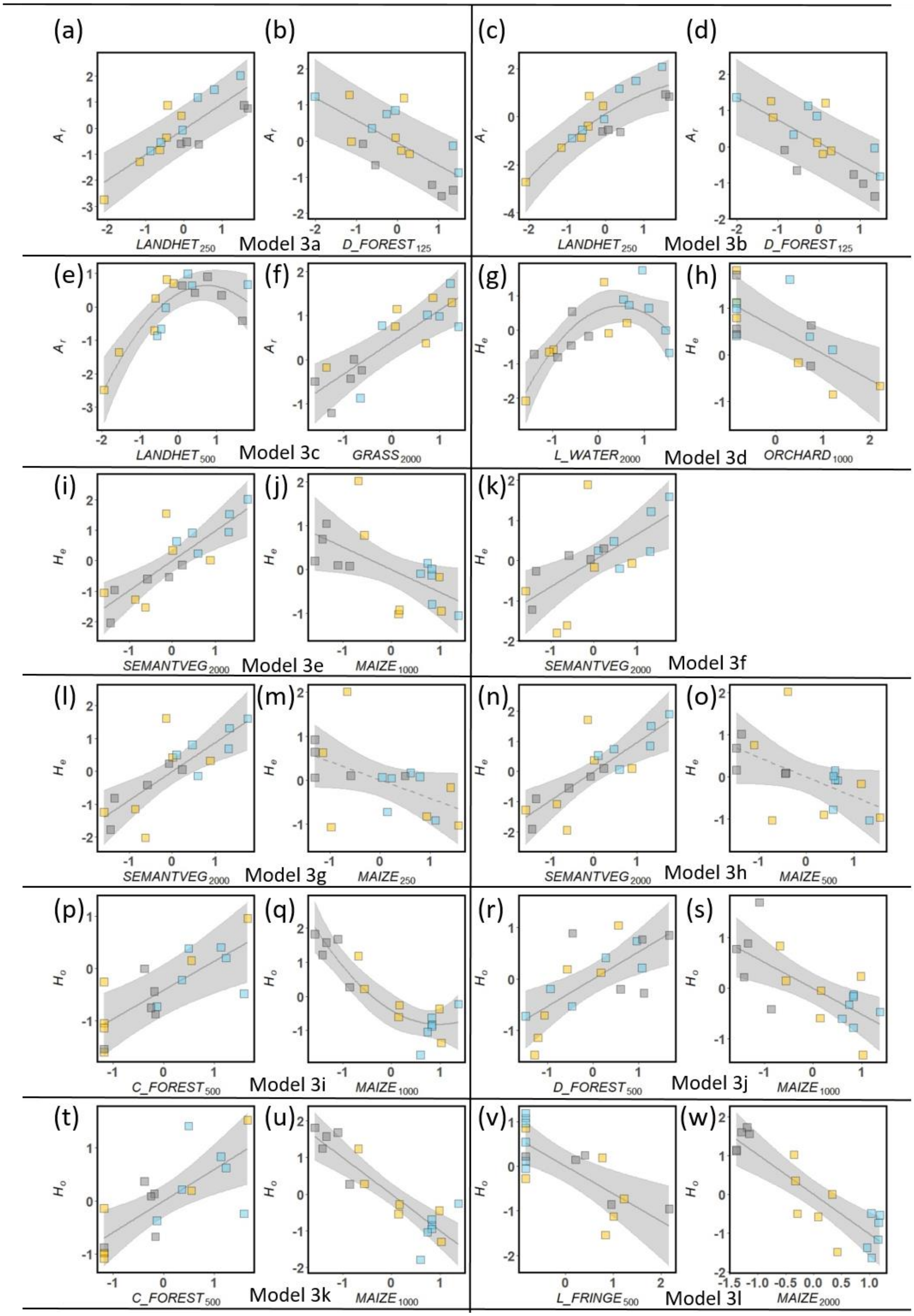

(a)
A_r
$LANDHET_{250}$
(b)
A_r
D_FOREST_{125}
Model 3a
(c)
A_r
$LANDHET_{250}$
(d)
A_r
D_FOREST_{125}
Model 3b
(e)
A_r
$LANDHET_{500}$
(f)
A_r
$GRASS_{2000}$
Model 3c
(g)
H_e
L_WATER_{2000}
(h)
H_e
$ORCHARD_{1000}$
Model 3d
(i)
H_e
$SEMANTVEG_{2000}$
(j)
H_e
$MAIZE_{1000}$
Model 3e
(k)
H_e
$SEMANTVEG_{2000}$
Model 3f
(l)
H_e
$SEMANTVEG_{2000}$
(m)
H_e
$MAIZE_{250}$
Model 3g
(n)
H_e
$SEMANTVEG_{2000}$
(o)
H_e
$MAIZE_{500}$
Model 3h
(p)
H_o
C_FOREST_{500}
(q)
H_o
$MAIZE_{1000}$
Model 3i
(r)
H_o
D_FOREST_{500}
(s)
H_o
$MAIZE_{1000}$
Model 3j
(t)
H_o
C_FOREST_{500}
(u)
H_o
$MAIZE_{1000}$
Model 3k
(v)
H_o
L_FRINGE_{500}
(w)
H_o
$MAIZE_{2000}$
Model 3l

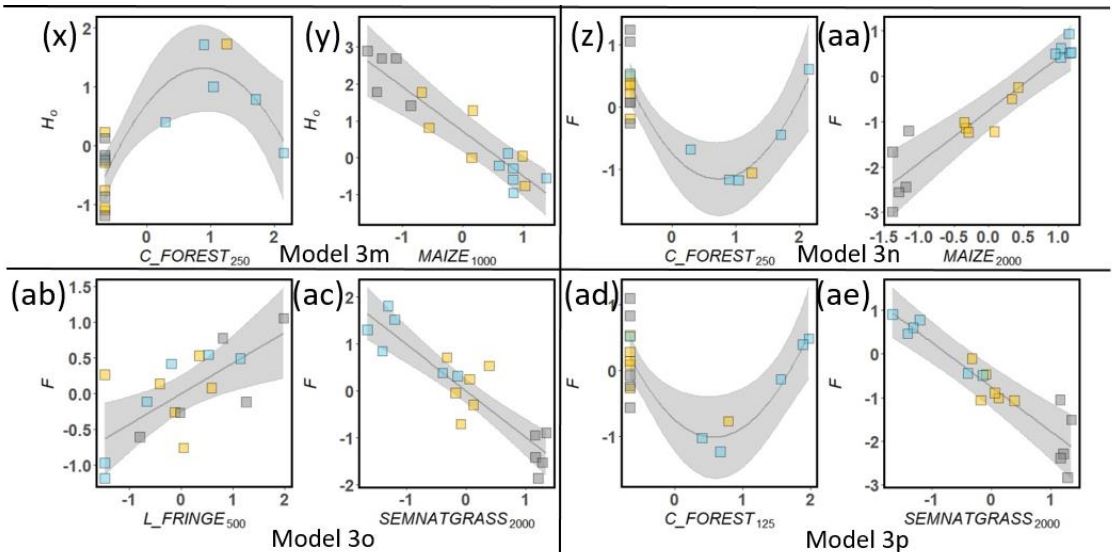

Figure S8.2: Visualization of landscape effects (cf. Table S8.2) on the population genetic structure of *P. multiflorum* (Step 3 Models best). Statistically significant effects ($p < 0.05$), are represented by solid lines and marginally significant effects ($p < 0.1$) by dashed lines with 95% confidence bands depicted in grey. Partial residuals are represented by filled squares. Due to the high number of Step 3 Models best at the link level, we only plotted the results for the node level.
