# Supplementary material for: Bumblebees mediate landscape effects on a forest herb's population genetic structure in European agricultural landscapes
Source: Ecol Evol. 2024 Jul 25;14(7):e70078. doi: 10.1002/ece3.70078 (PMC11269766; doi:10.1002/ece3.70078)
Supplement: Supplementary file 1 — Data S1. [file ECE3-14-e70078-s001.zip › README.docx]

**README for the R Data Analysis Script**

The script, designed for R version 4.0.4 and 3.6, performs a comprehensive data analysis following the four steps outlined in Figure 1 of the main text.

**Step 1: Landscape effects on genetic linker (1a. node level, 1b. link level)**

**A. Preparation**

Here, we merged the data sets containing bumblebee movement indicators and landscape metrics, including crop dominance values until 2019. Variables with too many zeros or missing values were excluded. Subsequently, we applied the Box-Cox transformation to all variables using the self-written function boxcox().

**B. Find best models for movement indicators**

In this step, we determined which landscape metrics significantly influenced the movement indicators in a single-metric model using the lme () function from the nlme package (Version 3.1-160). For the link level, we incorporated a dependency structure with the corMLPE () function from the corMLPE package (Version 0.0.3). We compared, for each land-use type, which buffer sizes or width-to-length ratios yielded the lowest *AICc* values. Additionally, we tested whether a unimodal model or a model including a quadratic term had a lower *AICc* for each landscape metric. The terms of the metrics with the lowest *AICc* were selected for further downstream analysis.

Next, we selected the landscape metrics whose single-metric models had *p*-values < 0.15. For the selection we tested whether two metrics covaried with |r| ≥ 0.7 or more.

All landscape metrics and movement indicators were standardized to have a mean of 0 and a standard deviation of 1. Subsequently, we run a full model (node level: LME; link level: MLPE) including all landscape metrics. We tested the residuals of this model for normality.

Next, we used the dredge() function of the MuMIn package (Version 1.47.1) to compare all models according to their *AICc*. We used specific conditions for the dredge-selection process:

- Models with a covariance |r| ≥ 0.7 were not permitted to be included in the same model.
- Quadratic terms for a landscape metric were only allowed if their unimodal term were also included in the model.

All models with delta *AICc* lower 2 were considered to be equally good. We ran all optimal models with delta AICc < 2 and tested the residuals of the models for normality.

**Step 2: Landscape effects on forest herb with landscape metric selected for movement indicators (2a. node level; 2b. link level)**

**A. Preparation**

In this step, we merged the data sets containing forest herb’s genetic structure measures and landscape metrics, including crop dominance values until 2017. Subsequently, we applied a Box-Cox transformation and standardized the data have a mean of 0 and a standard deviation of 1. The objects were saved for Step 3.

**B. Run Step 2 Models**

For all measures of forest herb’s genetic structure we run LMMs (Node level) and MLPEs (Link level). The models were saved for Step 4.

**Step 3: Running models for all possible combinations of landscape metrics (a. node level; b. link level)**

**A. Preparation**

For this step, we utilized Box-Cox transformed and standardized data frames obtained from Step 2. We generated all possible combinations of landscape metrics, filtering out combinations that included the same land-use types and those with a correlation greater than |r| ≥ 0.7.

**B. Run Step 3 Models**

Next, we ran all possible models and extracted their *AICc*-values. Due to the high number of combinations at the link level, we conducted this part of the analysis on the ZALF high-performance cluster (HPC). The total number of combination was divided into 20 subparts, and these subparts were concurrently processed on different nodes of the cluster. After completion, models with failed convergence using the lme() were subsequently removeds.

**Step 4: Comparison of the Step 2 Models and the Step 3 Models**

In this step, we calculated the percentage of *AICcs* of Step 3 models that were greater than the AICcs values of Step 2 models.
